# Psychometric Evaluation of the Altered States of Consciousness Rating Scale (OAV)

**DOI:** 10.1371/journal.pone.0012412

**Published:** 2010-08-31

**Authors:** Erich Studerus, Alex Gamma, Franz X. Vollenweider

**Affiliations:** 1 Neuropsychopharmacology and Brain Imaging and Heffter Research Center, University Hospital of Psychiatry Zurich, Zurich, Switzerland; 2 Department of Clinical and Social Psychiatry, University Hospital of Psychiatry Zurich, Zurich, Switzerland; 3 Neuropsychopharmacology and Brain Imaging and Heffter Research Center, University Hospital of Psychiatry Zurich, Zurich, Switzerland; King's College London, United Kingdom

## Abstract

**Background:**

The OAV questionnaire has been developed to integrate research on altered states of consciousness (ASC). It measures three primary and one secondary dimensions of ASC that are hypothesized to be invariant across ASC induction methods. The OAV rating scale has been in use for more than 20 years and applied internationally in a broad range of research fields, yet its factorial structure has never been tested by structural equation modeling techniques and its psychometric properties have never been examined in large samples of experimentally induced ASC.

**Methodology/Principal Findings:**

The present study conducted a psychometric evaluation of the OAV in a sample of psilocybin (*n* = 327), ketamine (*n* = 162), and MDMA (*n* = 102) induced ASC that was obtained by pooling data from 43 experimental studies. The factorial structure was examined by confirmatory factor analysis, exploratory structural equation modeling, hierarchical item clustering (ICLUST), and multiple indicators multiple causes (MIMIC) modeling. The originally proposed model did not fit the data well even if zero-constraints on non-target factor loadings and residual correlations were relaxed. Furthermore, ICLUST suggested that the “oceanic boundlessness” and “visionary restructuralization” factors could be combined on a high level of the construct hierarchy. However, because these factors were multidimensional, we extracted and examined 11 new lower order factors. MIMIC modeling indicated that these factors were highly measurement invariant across drugs, settings, questionnaire versions, and sexes. The new factors were also demonstrated to have improved homogeneities, satisfactory reliabilities, discriminant and convergent validities, and to differentiate well among the three drug groups.

**Conclusions/Significance:**

The original scales of the OAV were shown to be multidimensional constructs. Eleven new lower order scales were constructed and demonstrated to have desirable psychometric properties. The new lower order scales are most likely better suited to assess drug induced ASC.

## Introduction

Altered states of consciousness (ASC) represent a marked deviation in the subjective experience or psychological functioning of a normal individual from her/his usual waking consciousness [1]. As opposed to psychiatric diseases, ASC are short-lasting. They are usually self-induced (eg, by hallucinogenic drugs, meditation, hypnosis), but may also occur spontaneously in everyday life (eg, hypnagogic states). Although ASC are by definition different from psychiatric diseases, the study of ASC in healthy volunteers has a long tradition of generating hypotheses for psychiatric research.

Dittrich's APZ (Abnormal Mental States) questionnaire [1]–[4] and its revised versions, OAV [5] and 5D-ASC [6], [7], are among the most widely used self-report questionnaires for assessing subjective experiences of ASC in retrospect. Although originally developed in German, these questionnaires have been translated into many different languages and applied internationally in approximately 70 experimental studies. The majority of these studies have used these questionnaires to assess ASC induced by psycho-active drugs, particularly psilocybin (eg [8]), ketamine [9], MDMA (eg [10]), and *N,N*-dimethyltryptamine (DMT) (eg [11]), but several studies have also assessed non-pharmacologically induced ASC, such as ASC induced by endogenous psychosis [12], sensory deprivation [13], mind machines [14], and monochrome sounds [15]. The three versions of Dittrich's ASC questionnaires have been successfully applied to differentiate the subjective effects of different ASC induction methods [3], [16]); to characterize dose-response relationships [17]; and to map first person accounts of ASC to various neuronal, psychophysiological, and behavioral measures of ASC, including measures of positron emission tomography (PET) (eg [18]), functional magnetic resonance imaging (eg [9]), and electroencephalography (eg [19]).

The original version, APZ, contains 158 dichotomous items covering a broad range of phenomena potentially occurring during ASC. It was originally developed by Dittrich [1]–[4] in order to test the hypothesis that ASC – independent of their means of induction – have features in common that can be parsimoniously described on stable (ie, etiology-independent) major dimensions. Dittrich [3], [4] reasoned that if this hypothesis could not be falsified for a broad range of ASC induction methods, integration of phenomenological, psychophysiological, and neurobiological research on ASC would be greatly enhanced. For example, the detection of features that are common/invariant for all ASC and at the same time differentiate them from normal waking consciousness would help to lay the foundation for a more coherent definition of the term ASC. Furthermore, because these common features of ASC would remain indicators of the same underlying constructs across different ASC induction methods, ASC could be characterized and compared by their relative standing on stable etiology-independent latent dimensions. Moreover, establishing such dimensions would eventually lead to an empirical taxonomy of ASC.

Dittrich [3], [4] tested his hypothesis in a series of experimental studies, in which healthy volunteers were treated with one of eleven different ASC induction methods (*n* = 259) or by control condition procedures (*n* = 134). The studied induction methods were divided into four groups: (a) hallucinogens of the first order (ie, DMT, Psilocybin, and Δ^9^-tetrahydrocannabinol); (b) hallucinogens of the second order (ie, nitrous oxide); (c) sensory deprivation in a broader sense (ie, perceptual deprivation, hypnagogic states, autogenic training, hypnosis); and (d) sensory overload (ie, stimuli of high variety). From the 158 items of the APZ, which served as dependent variables, Dittrich [3], [4] identified 72 items meeting his criteria of etiology-independency. That is, these items had a significant proportion of yes-answers in each group of ASC induction methods and differentiated significantly between the treatment and control conditions. By analyzing the correlation matrices of the 72 etiology-independent items using exploratory factor and cluster analysis and based on considerations of stability, reliability, and interpretability, Dittrich [3], [4] determined three oblique primary and one secondary etiology-independent dimensions. The three primary dimensions were termed “oceanic boundlessness” (OBN), “dread of ego dissolution” (DED) and “visionary restructuralization” (VRS). The OBN scale basically includes items measuring positively experienced depersonalization and derealization, deeply-felt positive mood, and experiences of unity. High scores on the OBN scale therefore indicate a state similar to mystical experiences as described in the scientific literature on the psychology of religion (eg, see [20]). The DED scale includes items measuring negatively experienced derealization and depersonalization, cognitive disturbances, catatonic symptoms, paranoia, and loss of thought and body control. High scores on the DED scale therefore indicate a very unpleasant state similar to so called “bad trips” described by drug-users. The VRS scale contains items measuring visual (pseudo)-hallucinations, illusions, auditory-visual synesthesiae, and changes in the meaning of percepts. The secondary scale (G-ASC) consists of the 72 etiology-independent items and can be interpreted as a general measure of consciousness alteration. The validity of the experimental results in the field and the invariance of the factorial structure across different language versions of the APZ was examined and confirmed in a large international study on ASC [21], in which 1133 subjects from six different countries and four different languages completed the APZ in reference to their most recent ASC that they had experienced within the past 12 months.

Although reliabilities and validities of APZ scales were deemed to be acceptable in the experimental as well as in the field studies, several weaknesses were also recognized. For example, the binary item response format of the APZ was too crude to measure subtle alterations of consciousness. Furthermore, the OBN and VRS dimensions contained a relatively low number of items, and the conceptual breadth of the VRS dimension was considered too narrow. Bodmer et al. [5] therefore developed a psychometrically improved version called OAV. The abbreviation OAV stands for the German names of the three dimensions OBN, DED, and VRS. Because the OAV was supposed to measure the primary three dimensions of the APZ only, its item pool was primarily derived from 72 etiology-independent items of the APZ. However, the response format was changed from binary to visual analogue, several items were re-worded, some new items were introduced, and some items were completely dropped. The reformulation of items aimed not only at reducing cross-loadings, decreasing ambiguity, and enhancing ease of understanding, but also at widening the conceptual breadth of the OBN and VRS dimensions. Whereas the OBN dimension was changed toward a more complete assessment of mystical experiences by incorporating items that were formulated on the basis of six of the nine categories of mystical experiences proposed by Stace [20], the VRS dimension was conceptually widened by incorporating items that measure an increase of imaginations, associations, and memory retrieval. The re-conceptualization of the VRS dimension was mainly driven by theoretical considerations of Leuner [22], [23], who had hypothesized that visual hallucinations are associated with an increased internal stimulus production. The original OAV validation study [5], which was based on 177 subjects retrospectively describing their most recent ASC, indicated that the questionnaire revision successfully improved several psychometric properties, including item discriminations, simple structure and scale reliabilities. High correlations of OBN, DED, and VRS scales across the two questionnaire versions suggested that these scales measure similar constructs in both questionnaires. Results obtained by the APZ and OAV can therefore be compared by transforming the scales through linear equations [24].

Although the dimensional analyses of the APZ and OAV questionnaires had revealed three primary “etiology-independent” dimensions of ASC, Dittrich's own investigations [3], [4], as well as the scientific literature on ASC, pointed to the existence of further dimensions that are specific to certain ASC-inducing agents. For example, acoustic alterations and hallucinations are a common feature of ASC induced by certain psychiatric diseases, such as schizophrenia and alcohol withdrawal psychoses and have been described under conditions of sensory deprivation and hypnagogic states [25], but seem to be less common in hallucinogen-induced ASC [26]. In accordance with these findings, only 2 of the 11 APZ items measuring acoustic alterations met criteria of etiology-independency in Dittrich's experimental studies [3]. Furthermore, clouding of consciousness and reduction of vigilance are characteristic features of hallucinogens of the second order and of sedative drugs, but not of hallucinogens of the first order [23]. Dittrich [4], [16] therefore hypothesized that “auditory alterations” (AUA) and “vigilance reduction” (VIR) were two etiology-dependent dimensions, which, in addition to the three primary etiology-independent dimensions, could be reliably and validly measured. To test this hypothesis, Dittrich and co-workers constructed (Dittrich, Lamparter, Maurer and Schneiter, unpublished manuscript) and successfully validated (B. Schneiter, unpublished master's thesis) the so-called BETA (**Be**wüsstseins**t**rübung und **A**kustische Halluzinationen) questionnaire, which contains 17 and 22 items measuring the AUA and VIR dimensions, respectively. Because a dimensional analysis of the Pearson correlation matrix formed from the 39 BETA items and the 49 APZ items comprising the primary three scales indicated that the AUA and VIR dimensions could be differentiated from the OBN, DED and VRS dimensions and because the reliabilities and validities of the AUA and VIR scales were demonstrated to be acceptable (Braun, unpublished master's thesis), an extended version of the OAV, called 5D-ASC (“five dimensions of ASC questionnaire”) was published in 1999 [7], which includes 16 and 12 BETA items measuring the AUA and VIR dimensions, respectively. The 5D-ASC is the latest version of Dittrich's ASC questionnaires. Psychometrically yet untested versions of the 5D-ASC exist in U.S. English [7], French, Brazilian Portuguese, Arabic, Dutch and Japanese (A. Dittrich, personal communication, February 14, 2010).

Although Dittrich [1] concluded, that his original hypotheses on ASC have survived considerable falsification testing not only in experimental but also in field studies and that the APZ questionnaire has become a psychometrically well-validated instrument for the assessment of “aetiology-independent” features of ASC in a “aetiology-independent” three-dimensional space, it should be noted, that the studies carried out so far have serious methodological limitations, from which only few have been recognized in the existing literature. For instance, the dimensional analyses of the dichotomous APZ items were based on Pearson correlations among the items, which, unlike tetrachoric correlations, can be severely attenuated if the items differ markedly by their difficulties [27]. This is a significant problem because it may have led to the extraction of pseudofactors that reflect similar item difficulty rather than similar item content [28]. Another methodological shortcoming of Dittrich's original investigation is that the stability of the proposed factorial structure across different ASC induction methods and languages has only been examined by descriptive measures of factor pattern similarity derived from the comparison of EFA models, namely, by Tucker's coefficient of congruence and by Cohen's κ. These measures have many recognized problems [29]. For instance, because they only estimate the similarity of factor loadings, but not the similarity of indicator intercepts and residual variances, they can only provide evidence for the weakest forms of factorial invariance, namely, the so-called “metric invariance” or “weak factorial invariance” in the case of Tucker's coefficient of congruence and “configural invariance” in the case of Cohen's κ (or a description of different levels of factorial invariance, see [30]). However, even for the assessment of these weakest forms of factorial invariance, the use of these similarity measures is problematic, because their size is affected by various properties of the data [31], [32]. Thus, it is unclear how large they should be to conclude that factor pattern similarity holds to a reasonable degree. Commonly applied rules of thumb, which were also used in the study of Dittrich [1], [3], are not only unreliable, they also seem to be much too lenient [33]. Furthermore, due to his relatively low sample size, Dittrich [21] assessed the factor pattern similarity across different ASC induction methods only on the level of aggregated items and only across four groups of ASC induction methods. The use of item aggregates, however, is highly problematic when the goal is to represent the dimensionality of the measurement space at the level of individual items [34].

Another potential bias in Dittrich's original investigation is the use of a specific set of items. Analogous to the dimensions of personality (eg, the so-called “Big-Five”), broad dimensions of ASC can only be found by analyzing sets of items that are representative for the domain of interest. Although Dittrich [21] has originally derived his three primary dimensions of ASC from a set of 158 APZ items that were selected to be representative for the domain of interest, it is unknown whether the sampling of APZ items was indeed unbiased, because his investigation was never repeated in other independent sets of items.

Unfortunately, studies that have re-examined the psychometric properties of Dittrich's ASC rating scales after their first publication are scarce and those that exist were based on very limited sample sizes. Furthermore, because these rating scales were constructed and validated during the early 80s to the mid 90s, dimensional analyses have fully relied on exploratory methods. None of these scales has previously been analyzed by modern latent-variable approaches, such as confirmatory factor analysis (CFA) and structural equation modeling (SEM), which now have become standard methods of psychometric investigations and which are associated with many of the methodological and statistical advances in quantitative psychology in the last two decades [35]. Because these methods can also overcome many weaknesses of Dittrich's original investigations and more directly assess the validity of Dittrich's hypotheses, studies applying these methods on Dittrich's ASC questionnaires have long been overdue.

Another shortcoming of previous psychometric investigations is that they analyzed only major dimensions and did not explore potential lower order factors or so-called facets, even though applied researchers were not satisfied with the large conceptual breadth of the proposed major dimensions and have constructed their own subscales based on considerations of item content (eg [36]). Addressing these issues is important, because Dittrich's questionnaires continue to be widely used, and few validated instruments are available that measure similar subjective experiences. In fact, we are aware of only two instruments that measure similar experiences and that have gained similar acceptance in applied research, that is, the Phenomenology of Consciousness Inventory [37] and the Hallucinogen Rating Scale [38].

To overcome the methodological limitations of previous investigations, we performed a psychometric evaluation of the OAV in a relatively large sample of subjects describing experiences of ASC that were experimentally induced by psilocybin, ketamine, or MDMA. In contrast to previous studies, the factorial structure was explored and tested by using methods of the SEM-framework, including CFA and exploratory structural equation modeling (ESEM) [39], and by applying a hierarchical item clustering (ICLUST) [40] procedure that was specifically developed to display the hierarchical structure of a scale. This allowed us to investigate the factorial structure of the OAV not only on a dimensional level, but also on the level of lower order factors or facets. A number of lower order factors were extracted and compared with the original scales. The measurement invariance and population heterogeneity of these lower order factors across different drugs, settings, questionnaire versions, and sexes were examined by multiple indicators multiple causes (MIMIC) modeling. The reliabilities were assessed not only by Cronbach's α, but also by various non-standard reliability coefficients for both the original and newly-constructed scales. Furthermore, convergent, discriminant, and know-group validities of these scales were examined. The advantages of the newly constructed subscales, as well as the implications of our results with respect to Dittrich's original hypothesis, are discussed.

## Methods

### Ethics Statement

All pooled studies were approved by the Ethics Committee of the University Hospital of Psychiatry, Zürich, and the use of psilocybin, ketamine, and MDMA was authorized by the Swiss Federal Office of Public Health, Department of Pharmacology and Narcotics, Berne. All subjects gave their written informed consent prior to participation in the studies.

### Samples and Data Collection Procedures

The samples used in the present investigation were obtained by pooling data from 43 experimental studies (including pilot studies) carried out at our research facility between 1992 and 2008 involving psilocybin (115–350 µg/kg po), ketamine (6–12 µg · kg^−1^ · min^−1^ iv), or MDMA (1.5–1.7 mg/kg po) administration to healthy volunteers. The studies were part of a research program in which psilocybin, ketamine, and MDMA were used as tools for pharmacological modeling of core symptoms of schizophrenia and for investigating cognitive and perceptual processes [41], [42].

Participants of all studies were recruited through advertisement from the local universities and hospital staff. All subjects were carefully screened before admission to the studies. Subjects having personal or family (first-degree relatives) histories of major psychiatric diseases, neurological or substance related disorders, high emotional lability scores (more than two standard deviations above the normative mean in the Freiburg Personality Inventory [43]), or physical problems (according to a physical examination, electrocardiogram, and clinical-chemical blood test) were excluded. All drug sessions were performed by following safety guidelines that are similar to those recommended by Johnson et al. [44].

In each study, a placebo-controlled within-subject design was used. Depending on the study, subjects received placebo and 1–4 different doses or combinations of psychoactive drugs in 2–5 experimental sessions. Experimental sessions were conducted at least two weeks apart in order to avoid carry-over effects. In the majority of the studies (*n = *22), the order of drug administration was randomized and double-blind, but some of the earlier studies as well as most pilot studies (*n = 21*) were open-label trials. For the present investigation, we only used data from those experimental sessions, in which psilocybin, ketamine, or MDMA was administered alone. Very low dose psilocybin sessions (15–45 µg/kg po) were excluded due to statistically non-significant subjective drug effects [17]. In accordance with these criteria, psilocybin, ketamine, and MDMA were administered in 327, 162, and 102 experimental sessions, respectively. Racemic, (*R*)- and (*S*)-ketamine were administered in 6, 22, and 134 of the ketamine sessions respectively. The total sample consisted of 591 drug sessions. For a detailed description of the sample, see Table 1.

**Table 1:** Descriptive statistics.

	Psilocybin		Ketamine		MDMA		Combined		
Characteristic	*n = *327		*n = *162		*n = *102		*N = *591		Test statistic^a^
Age (*M* ± *SD*)	28.5±6.1		29.5±5.8		26.6±5.1		28.5±5.9		*F*(2, 588) = 7.7, *p*<.001
Gender									χ^2^ = 31.2, *p*<.001
Male	57%	(187)	80%	(130)	76%	(78)	67%	(395)	
Female	43%	(140)	20%	(32)	24%	(24)	33%	(196)	
Education									χ^2^ = 4.5, *p* = .345
High school diploma	7%	(24)	9%	(15)	5%	(5)	7%	(44)	
University students	35%	(116)	36%	(58)	28%	(29)	34%	(203)	
University graduates	57%	(187)	55%	(89)	67%	(68)	58%	(344)	
Dose									χ^2^ = 211, *p*<.001
Low^b^	22%	(72)	0%	(0)	0%	(0)	12%	(72)	
Medium^c^	65%	(214)	43%	(70)	100%	(102)	65%	(386)	
High^d^	13%	(41)	57%	(92)	0%	(0)	23%	(133)	
Questionnaire version									χ^2^ = 91.6, *p*<.001
5D-ASC	69%	(227)	26%	(42)	38%	(39)	52%	(308)	
OAV	31%	(100)	74%	(120)	62%	(63)	48%	(283)	
Setting									χ^2^ = 57.7, *p*<.001
PET	16%	(51)	48%	(77)	25%	(25)	26%	(153)	
No PET	84%	(276)	52%	(85)	75%	(77)	74%	(438)	

*Note.* Numbers in parenthesis indicate absolute frequencies. PET  =  positron emission tomography.

a Based on the comparison between the psilocybin, ketamine and MDMA groups. ^b^115 µg/kg psilocybin. ^c^215–270 μg/kg psilocybin, 1.5–1.7 mg/kg MDMA, 6 μg ⋅ kg^−1^⋅ min^−1^ ketamine. ^d^315 µg/kg psilocybin, 12 µg ⋅ kg^−1^⋅ min^−1^ ketamine.

Because some studies involved multiple drug sessions and because some subjects participated in more than one study, the above samples contain non-independent observations. Unfortunately, some of the multivariate statistical procedures used in the present study rely on the assumption of independency of observations. In order to control for this potential bias, we also analyzed samples that included only one experimental session per subject. For each subject we selected the experimental session that was conducted first. By applying these inclusion criteria, we obtained samples of the following sizes: psilocybin (*n = *186), ketamine (*n = *109), MDMA (*n = *95), and combined drug group (*n = *344).

### Measures

#### Altered states of consciousness rating scales (OAV and 5D-ASC)

In each experimental session, subjects were asked to describe the experiences of drug induced ASC by the German versions of the OAV or 5D-ASC questionnaires. The OAV was used in studies conducted before the year 2000 (*n = *27), while the 5D-ASC was used in all later studies (*n = *16). Because the 5D-ASC is an extension of the OAV, all 66 OAV items are also fully contained in the 5D-ASC. They also appear in the same order in both questionnaires, but are interspersed by 5D-ASC unique items when presented to the subjects as part of the 5D-ASC. Because the available samples would have been too small to investigate the factorial structures of both questionnaires, items from the 5D-ASC data were combined with the corresponding items of the OAV in the present study. Each OAV item contains a statement describing a specific experience of ASC in the past tense (eg, “It seemed to me that my environment and I were one”). Subjects were instructed to respond to the described experiences by placing marks on horizontal visual analogue scales (VAS) of 100 millimeters length. The VAS of the OAV are anchored as *no, not more than usual* on the left and as *yes, very much more than usual* on the right. The items are scored by measuring the millimeters from the low end of the scale to the subject's mark (integers from 0–100). Because the low end of the scale indicates a neutral response, the response format of these items can be considered as *strictly unipolar* according to the response format typology of Russel and Carroll [45].

In most studies, the OAV and 5D-ASC were completed during or shortly after the drug effects peaked. However, in some studies, these rating scales were completed after the drug effects had worn off or at multiple time points. In the latter case, we only included those measures that were obtained during the peak drug effect. Depending on the study, the pooled OAV and 5D-ASC questionnaires were completed 60–300 min after psilocybin, 25–120 min after ketamine, and 70–160 min after MDMA administration. Subjects were instructed to retrospectively rate their whole experience from the moment of drug intake to the respective measuring time point.

#### Short Version of the Adjective Word List (“Eigenschaftswörterliste”; EWL-60-S)

The EWL-60-S [46] is a German self-report rating scale for the multidimensional assessment of the current mental state. It is composed of a list of 60 adjectives (eg, “anxious”, “tired”, “sociable”), which can be grouped into 15 subscales each comprising 4 adjectives (see Table 6 for the names of these subscales). The subscales can be further grouped into six domains. Subjects are asked to respond to the adjectives on four-point Likert scales ranging from 0 (*not at all*) to 3 (*very much*). The EWL-60-S is a short version of the original EWL-N and -K questionnaires [47], which have a very similar factorial structure but use a dichotomous instead of a Likert-type response format. The EWL-60-S has been found to be well suited to measure short-term changes of mental states induced by psychoactive drugs (eg [10]), psychological stress [48], and embodying of emotion [49]. Internal consistencies (Cronbach's α) for the subscales of the EWL-60-S were reported to range between 0.40 and 0.86 in a sample of elderly people (*n* = 128) and between 0.72 and 0.91 in a student sample (*n* = 67) [46]. The validity of the EWL-60-S has been mostly inferred from the validity studies of the EWL-N and –K, from which most of the EWL-60-S items were taken.

**Table 2:** Confirmatory factor analysis model fit result.

	df	MLR χ^2^	CFI	TLI	RMSEA	SRMR	AIC	Δdf	Δχ^2^	p
**Bodmer**'**s original structure**										
CFA (simple structure)	2076	6661.7	.709	.699	.061	.094	363480			
CFA with method effects	2069	6190.6	.738	.729	.058	.089	362819	7	393	<.001
ESEM	1950	5469.9	.777	.754	.055	.050	150706	119	670	<.001
ESEM with method effects	1943	5235.3	.791	.769	.054	.054	150103	7	37	<.001
Bifactor model	2013	5487.6	.779	.765	.054	.086	150545	−70	−223	
Bifactor model with method effects	2006	5116.9	.803	.789	.051	.095	150061	7	942	<.001
OBN factor alone	324	1643.7	.785	.767	.083	.070	64550			
DED factor alone	189	612.5	.834	.815	.062	.070	42944			
VRS factor alone	135	1199.5	.689	.648	.116	.090	45175			
**Model revision**										
Initial ICLUST solution (47 items)		2016.0	.907	.897	.042	.059	255679			
Final model (42 items)		1430.8	.929	.921	.038	.052	94687			
**MIMIC models**										
Final model: MIMIC without DIF		1780.6	.918	.904	.040	.050	97624			
Final model: MIMIC with DIF		1668.3	.928	.915	.038	.048	97489	9	120	<.001

*Note.* MLR  =  maximum-likelihood-robust estimator; CFI  =  comparative fit index; TLI  =  Tucker-Lewis index; RMSEA  =  root mean square error of approximation; SRMR  =  standardized root-mean-square residual; AIC  =  Akaike's information criterion; Δχ^2^  =  Satorra-Bentler scaled χ^2^ difference; CFA  =  confirmatory factor analysis; ESEM  =  exploratory structural equation model; OBN  =  oceanic boundlessness; DED  =  dread of ego dissolution; VRS  =  visionary restructuralization; ICLUST  =  hierarchical item-clustering; MIMIC  =  multiple indicators multiple causes; DIF  =  differential item functioning.

In the present investigation, the EWL-60-S was used to assess the convergent and discriminant validities of the OAV scales. The EWL-60-S was administered in 10 of the 43 pooled studies and in 177 of the 591 analyzed drug sessions. These drug sessions mostly involved the administration of psilocybin (*n* = 128) and less frequently of (*S*)-ketamine (*n* = 33) and MDMA (*n* = 16). In cases where the EWL-60-S was administered at multiple time points during one drug session, we used only those measures that were obtained during the peak drug effects. The internal consistencies (Cronbach's α) of the EWL-60-S subscales in our sample were mostly good to excellent and ranged from 0.76 to 0.91.

#### The State-Trait-Anxiety Inventory – State version (Form X; STAI-S)

The STAI-S [50] (German translation by [51]) is a very popular self-report rating scale designed to measure transitory feelings of tension and apprehension, or state anxiety. It contains 10 items describing symptoms of anxiety (eg, “I feel nervous”) and 10 items describing the absence of anxiety (eg, “I feel calm”). The German translation of the STAI-S has shown excellent internal consistency (average α≈.90) and adequate convergent and discriminant validities with scales of the original EWL questionnaire [51]. Furthermore, the revised English version of the STAI-S (Form Y) has demonstrated high sensitivity for the detection of stress [52]. However, despite these generally positive psychometric properties, the STAI-S has been criticized for its inability to adequately discriminate between symptoms of anxiety and depression [53], [54] and for its lack of unidimensionality. Most of the studies investigating the dimensionality of the STAI reported results indicating that the STAI-S scale could be further divided on the basis of whether the items were keyed in the direction of the presence or absence of anxiety [55]. Consistent with the view that state-anxiety is better accounted for by two unipolar instead of one bipolar construct, the state anxiety present and state anxiety absent scales have been shown to be differentially affected by situational [56] and cultural [57] variables.

Because the OAV contains subscales tapping symptoms of anxiety as well as the absence of anxiety/well-being, the three STAI-S scales (total scale, anxiety present, and anxiety absent/calmness) were used to assess convergent and discriminant validities of the OAV scales. The STAI-S was concurrently administered with the OAV in 56 of the pooled experimental drug sessions, 45 of which were MDMA and 11 of which were psilocybin sessions. All three subscales showed good internal consistencies in our sample (total scale: α = 0.88, anxiety present: α = 0.82, anxiety absent: α = 0.84).

### Statistical Analysis

The originally hypothesized factorial structure of the OAV was tested by CFA and ESEM [39], [58] using M*plus* Version 5.2 [59]. ESEM is a recent statistical development currently only available in M*plus* that integrates many advantages of exploratory factor analysis (EFA) and CFA by including an EFA measurement model part into a SEM framework. In the present study, we complemented the CFA with ESEM because the imposed simple structure of CFA models, that is, constraining non-target factor loadings to zero, is often inappropriate when analyses are done at the item level and when there are multiple factors, each measured with a reasonable number of items [35]. Furthermore, ESEM allowed us to perform an EFA while at the same time having full access to all the usual SEM parameters and also taking method effects into account, which may have resulted from items sharing similar wording. Whereas in conventional EFA, method effects can confound the detection of more meaningful factors, they can be controlled in ESEM by allowing correlated residuals [39].

In order to more fully explore the adequacy of the hypothesized three-dimensional solution, the appropriate numbers of factors to extract was examined by means of Cattell's scree test [60], Horn's parallel-analysis [61], Velicer's minimum average partial (MAP) test [62], Revelle's Very Simple Structure (VSS) Criterion [63], and Revelle's hierarchical item clustering (ICLUST) algorithm [40] using functions provided by the nFactors- [64] and psych-packages [65] of the statistical software R [66].

Because a well fitting simple structure CFA model with clearly defined factors, that is, factors that were measured by at least 3 items and that were conceptually meaningful, was impossible to achieve by the traditional EFA approach and by retaining all 66 OAV items in the solution even when the number of factors was greatly increased, we used cluster analysis as an alternative heuristic for initial CFA model specification. Although rarely used in applied research, a simulation study by Bacon [67] suggests that cluster analytic approaches to initial model specification are valuable alternatives to the more conventional EFA-related approaches, because they may lead to better fitting initial CFA models, which in turn reduces the need for extensive CFA model refinement and consequently the dangers of so-called specification searches.

We applied Revelle's ICLUST procedure, a cluster analytic approach that was specifically developed to cluster questionnaire items and that was recently implemented in the freely available psych-package [65] of the software R. ICLUST hierarchically clusters items using correlations corrected for attenuation as a proximity measure and the size of the reliability coefficients Cronbach's α and Revelle's β [68] as stopping rules. A major advantage of ICLUST is that items are only added to clusters if they increase the cluster's internal consistency and factorial homogeneity. Furthermore, as the sequential item-by-item growth of clusters mapped with an accompanying set of homogeneity statistics can be displayed in a hierarchical tree diagram, the ICLUST procedure provides uniquely useful diagnostic and interpretative information not available in conventional approaches of scale construction, such as EFA [69]. For instance, the internal substructure of scales can be directly visualized, and defensible decisions can be made on whether to form scales on a macro level (higher order scales) and at a more finely grained micro level (lower order scales). Because problematic items usually get merged in a late step of the ICLUST procedure, they can be more easily identified and they do not obscure the factorial structure as much as in an EFA (for more information, see [69]).

An initial simple structure CFA model with correlated latent factors was specified and evaluated on the basis of ICLUST item clusters meeting the following criteria: Satisfactory indexes for internal consistency (Cronbach's α>0.8) and homogeneity (Revelle's β>0.7), a minimal cluster size of three items, good interpretability, and conceptual importance. The initial factorial solution was then further refined by dropping items with high cross-loadings.

After having established a well fitting CFA model in the total sample, we used MIMIC modeling to examine population heterogeneity and differential item functioning (DIF) across different drugs, questionnaires, settings, and sexes. Although the multiple-groups CFA approach is more commonly used to examine structural and measurement invariance, we decided to use MIMIC modeling, because it requires lower sample sizes and allows the simultaneous evaluation of many different contrast variables [70]. Whereas multiple-groups CFA entails the simultaneous analysis of two or more measurement models, MIMIC involves a single CFA model in which latent factor and item indicators are regressed on covariates. A significant direct effect of a covariate on a latent factor indicates that the mean of the latent factors differs across different levels of the covariate (also referred to as population heterogeneity). A significant direct effect of a covariate on an observed item indicator is evidence for measurement non-invariance, because it means that the item endorsement is significantly different across different levels of the covariate even though the latent factor is held constant. The occurrence of measurement non-invariance (also referred to as DIF) in MIMIC corresponds to the occurrence of non-invariant item intercepts in multiple-groups CFA and has important consequences for the interpretation of latent factor means. That is, if DIF is present, group comparisons of latent factor means are confounded by group differences in the factor structure and therefore cannot be meaningfully interpreted unless group comparisons are made within the SEM framework, where DIF can be accounted for [70].

In the present study, we first examined a MIMIC model in which only the latent factors were regressed on the covariates. Because all direct effects between the covariates and the items were fixed to zero, this constituted the no-DIF model. The latent factors were regressed on the three binary variables female (0 =  male, 1 =  female), PET (0 =  experimental session involved no PET, 1 =  experimental session involved PET), and OAV (0 =  5D-ASC, 1 =  OAV) and the three-level nominal variable drug. The variable drug was represented in the model as two dummy coded contrast variables using the MDMA group as the reference group. For each of the five binary variables included in the MIMIC model, the minority or focal group contained at least 100 cases. A recent simulation study [71] suggests that focal groups of this size are large enough to produce reasonably powerful and accurate MIMIC results when the sample size is similar to our study.

To detect differential functioning (D–F) items we used the so-called “free baseline designated anchor approach” (for applied examples, see [72], [73]), which is supposed to have a lower false discovery rate than the more commonly applied stepwise forward procedure (eg [70]) and is most similar to well tested item response theory based methods [71]. The procedure involved two steps. First, anchor items were identified by regressing one item at a time on the five grouping variables (while constraining all other direct effects to zero) and testing the five regression parameters for significance. Items with no significant regression parameters were defined as DIF-free or anchor items. In the second step, all items not included in the DIF-free subset were tested for DIF by using likelihood ratio (LR) difference tests for nested models. That is, for each studied item, a comparison was made between a full model (all items were allowed to have DIF except for the anchor items) and a more constrained model (all items were allowed to have DIF except for the anchor items *and* the studied item). If the model fit of the constrained model was significantly worse relative to the full model, it was concluded that the studied item had DIF. As recommended by Woods [71], *p-*values of LR difference tests were adjusted by the Benjamini-Hochberg procedure [74] using the p.adjust function in R to control the false discovery rate. After all D–F items were identified, a model was fitted in which − in addition to the latent variables − only D–F items were regressed on the grouping variables. However, to further increase model parsimony, direct effects that were non-significant at *p<*0.05 or had very low effect sizes (y-standardized regression coefficients <0.2) were dropped in the final MIMIC model.

Because most OAV items were positively skewed (mean  = 1.25, range  = −0.56 to 4.32) and kurtotic (mean  = 1.27, range  = 1.64 to 19.23) and because our data set contained non-independent observations, latent factor models (CFA, ESEM, and MIMIC) were fitted by using the Robust Maximum Likelihood (MLR) estimator in combination with the “Complex” option in M*plus*. This method produces adjusted standard errors and fit indexes for non-normal and clustered data by means of a sandwich estimator and the Yuan-Bentler *T*_2_ * test statistic [59], [75]. Because the χ^2^ statistic of the MLR estimator cannot be used for χ^2^ difference tests, the Satorra-Bentler scaled χ^2^ difference test [76] was used for the comparison of nested models.

Unfortunately, most OAV items were not only positively skewed and kurtotic, but also showed a strong piling up of values at the lower end of the scale (39% of zero values on average and 71% at the most) and a modest piling up of values at the upper end of the scale (6% of values on average and 27% at the most; see Table S1 for the distributional characteristics of each item). Because parameter estimates produced by MLR can be biased to some degree if strong floor- or ceiling-effects are present, we cross-checked our results by categorizing the OAV items into 5 categories and using the polychoric correlation matrix calculated from categorized variables as input for the latent factor models and the ICLUST procedure (see Table S2 for the distributional characteristics of the categorized items and Methods S1 for more details on polychoric correlations and categorical data analysis).

Because the χ^2^ test of exact model fit is strongly influenced by sample size, adequacy of fit of the latent factor models was evaluated by Bentler's comparative fit index (CFI), the Tucker-Lewis index (TLI), and the RMSEA. Additionally, the standardized root mean square residual (SRMR) was used for models with continuous outcomes, and the weighted root mean square residual (WRMR) was used for models with categorical outcomes. In line with recommendations of Hu and Bentler [77], CFI and TLI values close to .95 or greater, RMSEA values close to .06 or below and SRMR close to .08 or below were considered as indicating reasonably good model fit. For the WRMR, a cut-off value close to 1.0 or below was considered suitable.

Because the newly constructed OAV scales did not meet the assumption of essential tau-equivalence (ie, equality of factor loadings) and because the original OAV scales additionally were non-congeneric, that is, they contained several group factors in addition to a general factor, we did not primarily rely on Cronbach's α for assessing scale reliability. Although α is the most commonly used reliability estimate, it has long been pointed out by several authors that α is not a dependable estimator of scale reliability when the above assumptions are not met and that several alternative reliability estimates exist that obviate the difficulties encountered with the use of α and that can be more easily interpreted [68], [78], [79]. In the present study, scale reliabilities of the newly constructed scales, which had been shown to be congeneric in the CFA, were directly derived within the SEM framework by using an approach described by Raykov [80], [81]. Point estimates of these reliability estimates, hereinafter referred to as ρ_SEM_, were supplemented by confidence intervals found by the so-called delta-method (eg, see [82]) to gain ranges of plausible values for the population scale reliabilities. For the original scales not meeting the assumption of unidimensionality, scale reliability was mainly assessed by using McDonalds ω_H_ and ω_T_ [78], [83]. Whereas ω_H_ estimates the amount of variance in a scale attributable to one common factor, also referred to as general factor saturation, ω_T_ estimates the amount of variance due to all common factors (ie, group factors *and* general factor). As we had no clear expectations regarding the number of group factors present in the original OAV scales as well as regarding the patterns of the loadings on the common factors, ω_H_ and ω_T_ were estimated by performing a higher order EFA analysis using the omega function of the psych-package [65] in R. This method has shown good performance in a simulation study [84]. For each scale, the number of group factors to extract was determined by parallel analyses [61]. As an alternative estimate of the general factor saturation and as an index of homogeneity, we also computed Revelle's β [68] using the ICLUST function in the psych-package. Furthermore, Cronbach's α was calculated in order to compare our results with the standard estimate of scale reliability and with the results of older studies. Confidence intervals for α were calculated using the method described by Duhachek and Iacobucci [85].

Criterion validities of the original and newly constructed OAV scales were evaluated by assessing convergent and discriminant validities, as well as known-group validities. Convergent and discriminant validities were assessed by correlating the OAV scales with subscales of the EWL-60-S and the STAI-S subscales. Known-group validities were examined by comparing the mean OAV scale scores of the three drug groups.

## Results

In contrast to our hypothesis, associations between items of opposite affective valence with L-shaped bivariate distributions and between latent constructs measuring opposite affective valence did not become more negative when estimated by polychoric instead of product-moment correlations. In fact, polychoric correlations were almost always more positive than product-moment correlations. The difference between the two estimation methods was 0.11 on average. However, because the factorial solution resulting from the analysis of categorized OAV items did not markedly differ from the factorial solution of the continuous items, except that the correlations between the latent variables were generally larger, we only report results from those statistical analyses that treated the OAV items as continuous variables. Results from analyses based on categorized variables are available upon request from the first author.

### Fit of the Originally Hypothesized Model

Table 2 provides fit indexes from a series of latent factor models testing Bodmer's originally hypothesized factorial structure of the OAV. When modeled as simple structure CFA with no correlated residuals and unconstrained latent factor covariances, Bodmer's factorial structure did not fit the data well. Although the parsimony-adjusted RMSEA and the absolute fit index SRMR were only slightly above the recommended cutoffs, comparative fit indexes (ie, CFI and TLI) were clearly unacceptable. Large modification indexes for the residual covariances between the items 8, 13, 20, and 25 and between the items 14 and 51, as well as similar wordings within these two item clusters, suggested that item covariances within these two item clusters might be explained in part by shared method effects. We therefore specified a less constrained CFA model in which residual covariances between the items 8, 13, 20, and 25 and between the items 14 and 51 were allowed to freely co-vary. Although this model fitted significantly better than the original model according to the scaled χ^2^-difference test, comparative fit indexes were still clearly unacceptable. Because the imposed simple structure of a standard CFA model is often unnecessarily restrictive [35], we next tested geomin- and quartimin-rotated 3-Factor-ESEMs with and without method effects. As expected, the free estimation of cross-loadings significantly improved model fit. Because all available fit indexes, including the parsimony-adjusted RMSEA, improved, the increase in model fit was not primarily achieved at the expense of increased model complexity. However, even when method effects were taken into account, overall model fit was relatively poor as the CFI and TLI values were still far below their recommended cutoffs. An inspection of the item loadings of the geomin-rotated ESEM without method effects revealed that 59 of the 66 items (89.4%) had their highest loading on the hypothesized factors. Six VRS items (# 17, 18, 37, 40, 52, and 64) describing experiences of changed meaning of percepts, facilitated recollection, and insightfulness loaded highest on the OBN factor and one VRS item (# 58) describing experiences of macropsia and micropsia loaded highest on the DED factor. Although all items had at least one significant main factor loading of at least modest size (>0.3), 28 items demonstrated also significant cross-loadings. The geomin-rotated ESEM that included method effects showed a considerably different pattern of factor loadings. In this model, only 54.5% of the items were correctly distributed to their hypothesized factors. Whereas the OBN and VRS factors essentially collapsed into one large first factor, the DED factor was divided into one factor tapping experiences of anxiety and another factor tapping experiences of impaired control and cognition. Tables S3 and S4 show the hypothesized and empirical item distributions resulting from the geomin- rotated 3-Factor-ESEMs with and without method effects, respectively. Quartimin-rotated ESEMs only marginally differed from their geomin-rotated counterparts.

**Table 3:** Correlations between latent factors and observed correlations of the OAV.

Scale	Experience of unity	Spiritual experience	Blissful state	Insightfulness	Disembodiment	Impaired control and cognition	Anxiety	Complex imagery	Elementary imagery	Audio-visual synesthesiae	Changed meaning of percepts
**New scales**											
Experience of unity	–	.82	.72	.78	.74	.50	.28	.61	.41	.54	.65
Spiritual experience	.69	–	.67	.80	.53	.27	.24	.53	.37	.42	.47
Blissful state	.62	.53	–	.63	.39	.11	−.07	.45	.25	.34	.46
Insightfulness	.63	.61	.49	–	.49	.34	.30	.61	.45	.47	.67
Disembodiment	.64	.45	.35	.39	–	.64	.37	.49	.33	.44	.48
Impaired control and cognition	.43	.24	.11	.27	.53	–	.70	.43	.36	.35	.62
Anxiety	.25	.22	−.06	.24	.32	.63	–	.23	.26	.20	.39
Complex imagery	.52	.44	.39	.48	.40	.36	.20	–	.73	.65	.53
Elementary imagery	.37	.34	.25	.36	.29	.33	.27	.62	–	.70	.41
Audio-visual synesthesiae	.49	.38	.32	.39	.39	.31	.18	.56	.62	–	.44
Changed meaning of percepts	.55	.39	.40	.53	.38	.50	.32	.42	.35	.38	–
**Old scales**											
Altered state of consciousness	.83	.66	.57	.68	.70	.69	.51	.70	.62	.64	.72
Oceanic boundlessness	.92	.76	.76	.71	.72	.46	.25	.58	.43	.52	.62
Dread of ego dissolution	.39	.26	.03	.30	.50	.91	.86	.34	.35	.29	.48
Visionary restructuralization	.62	.50	.46	.61	.47	.49	.32	.82	.79	.78	.69

*Note.* Correlations between latent factors are above the diagonal. Correlations based on scale sum scores are below the diagonal. All correlations were statistically significant at *p*<.001 except for blissful state with dread of ego dissolution, anxiety, and impaired control and cognition.

Because previous EFAs of the APZ and OAV had revealed large first eigenvalues relative to later eigenvalues and because the existence of a general factor has been hypothesized for both the APZ and OAV [5], [21], we also tested a bi-factor model in which all items were allowed to load on a general factor in addition to their specific group factor. Although this model fitted better than all previously tested models, comparative fit indexes were still clearly unacceptable. In order to examine the homogeneity of the hypothesized factors, we also modeled each factor separately. The results indicated that none of the three hypothesized factors can be considered unidimensional and that VRS is the most heterogeneous factor.

### The Optimal Number of Factors to Extract

Although the OAV questionnaire was specifically designed to measure three dimensions of ASC, none of the methods that we used to determine the optimal number of factors to extract indicated a three-dimensional solution. Parallel analysis, which is considered as one of the most effective and accurate methods for determining the number of factors to retain [86], suggested 5 and 13 factors, depending on whether the analysis was based on principal component (PA-PCA) or principal factor (PA-PFA) eigenvalues, respectively. In case of the PA-PFA, the number of factors was reduced to 11, when the observed eigenvalues were compared with the 95^th^ percentiles instead of the means of the eigenvalues generated from random data. Although in a recent Monte Carlo study [87], PA-PFA outperformed PA-PCA under conditions similar to our study (presence of correlated factors and strong general factor saturation as well as group factors), the scree test supported the results of the PA-PCA by also suggesting a five-factorial solution. However, the MAP-test indicated seven factors to retain, while the VSS criterion for complexity one and two favored one- and two-factorial solutions, respectively. Furthermore, the ICLUST algorithm, which clusters scales as long as the homogeneity and internal consistency of the higher level scale is greater than that of either subcomponent, did not stop until two clusters were left. One of these two item clusters comprised all DED items, while the other comprised all OBN and VRS items. Finally, by testing the fit of ESEMs with a varying number of factors, it was determined that at least 11 factors were necessary to achieve acceptable overall model fit. The optimal numbers of factors obtained by the methods discussed above are summarized in Table S5.

### Construction of new OAV Scales

**Figure 1:**
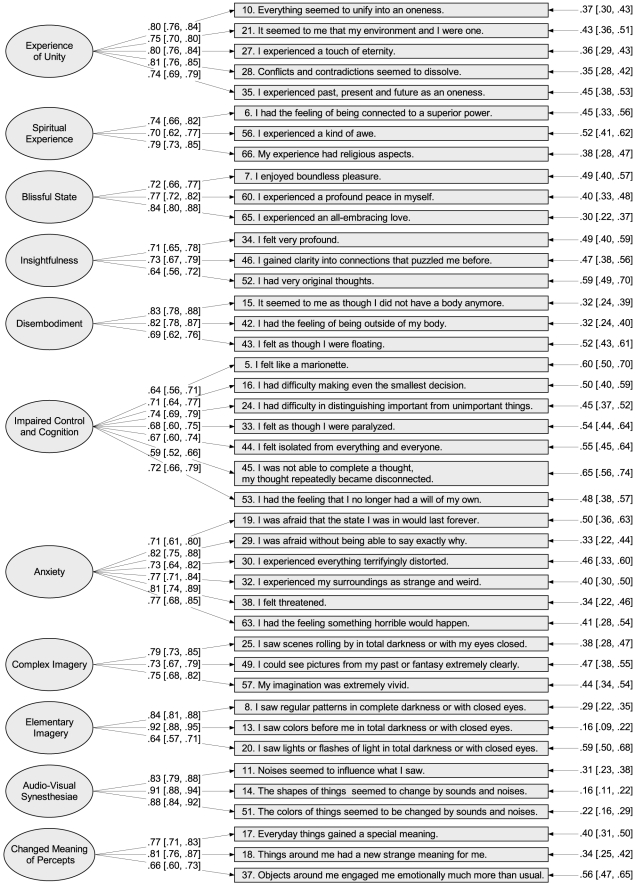
Final confirmatory factor analysis model with completely standardized loadings and error variances. Numbers in brackets are the 95% confidence intervals for the estimates. Covariances between factors were freely estimated and are shown in Table 3.

Although ESEMs with 11 or more factors fit reasonably well, they did not serve well as a basis for initial CFA model specification, because they contained several poorly defined factors and a relatively large number of items with significant cross-loadings (see Table S6 for the loading matrices of ESEMs with an increasing number of factors starting with the originally hypothesized three-factor solution). Instead of dropping multi-dimensional items step by step and thereby using CFA in an exploratory fashion, which is generally not recommended, because it can lead to problematic specification searches [70], we inspected the tree diagram produced by ICLUST to directly derive homogeneous and reliable subscales. By applying the criteria defined in the method section, 11 item clusters formed from 47 of the 66 original items were detected and used for initial CFA model specification. The ICLUST tree diagram and the item clusters that were used for the initial CFA model are shown in Fig. S1 (for the ICLUST tree diagram based on the categorized variables see Fig. S2). As the model fit of the initial CFA model was not sufficient according to the CFI and TLI indexes (see Table 2), we tried to improve model fit by dropping items showing large modification indexes for cross-loadings and ambiguous item wordings. The model revision led to a final model that still contained the same number of factors, but a slightly lower number of items (42 instead of 47). Because the dropped items (# 12, 39, 41, 48, and 54) had been mostly assigned to different factors, the model revision did not lead to a major change in the interpretation of any factor. Figure 1 shows the factorial structure of the final model, including the names that we gave to the 11 factors and the fully standardized loadings and error variances. The correlations between the latent factors as well as their associations with the original OAV scales are shown in Table 3. Although the CFI and TLI of this final CFA model were still slightly below the recommend cutoffs, the RMSEA and the SRMR indicated excellent model fit (see Table 2).

**Table 4:** Y-standardized regression coefficients [and 95% confidence intervals] of the final MIMIC model with DIF.

Factor	PET	Female	OAV	Psilocybin	Ketamine
Experience of unity	**0.27**	**0.25**	0.12	0.13	0.21
	[0.06, 0.47]	[0.03, 0.47]	[−0.09, 0.34]	[−0.14, 0.39]	[−0.07, 0.48]
Spiritual experience	**0.26**	0.09	**0.27**	**0.43**	**0.31**
	[0.04, 0.49]	[−0.18, 0.36]	[0.04, 0.50]	[0.16, 0.70]	[0.05, 0.58]
Blissful state	0.20	0.06	**0.28**	−0.27	**−0.79**
	[−0.02, 0.41]	[−0.16, 0.27]	[0.08, 0.48]	[−0.54, 0.01]	[−1.06, −0.52]
Insightfulness	**0.30**	−0.17	**0.32**	**0.60**	0.24
	[0.08, 0.52]	[−0.40, 0.06]	[0.07, 0.56]	[0.33, 0.88]	[−0.04, 0.52]
Disembodiment	**0.53**	**0.25**	0.03	0.14	**0.75**
	[0.33, 0.72]	[0.04, 0.46]	[−0.17, 0.23]	[−0.09, 0.37]	[0.51, 0.99]
Impaired control and cognition	**0.31**	**0.36**	−0.13	**0.26**	**0.66**
	[0.10, 0.52]	[0.11, 0.60]	[−0.34, 0.08]	[0.02, 0.49]	[0.40, 0.91]
Anxiety	**0.31**	0.05	0.04	**0.28**	**0.31**
	[0.09, 0.53]	[−0.15, 0.25]	[−0.15, 0.22]	[0.10, 0.46]	[0.08, 0.54]
Complex imagery	**0.45**	0.15	0.02	**0.80**	**0.41**
	[0.24, 0.66]	[−0.08, 0.38]	[−0.20, 0.23]	[0.54, 1.05]	[0.13, 0.69]
Elementary imagery	**0.36**	0.03	0.08	**1.44**	**0.73**
	[0.19, 0.53]	[−0.15, 0.21]	[−0.10, 0.26]	[1.28, 1.61]	[0.53, 0.93]
Audio-visual synesthesiae	−0.04	0.05	0.19	**0.87**	**0.55**
	[−0.23, 0.14]	[−0.17, 0.27]	[−0.02, 0.39]	[0.68, 1.06]	[0.34, 0.75]
Changed meaning of percepts	**0.28**	−0.06	**0.33**	**0.44**	−0.17
	[0.07, 0.49]	[−0.32, 0.21]	[0.11, 0.55]	[0.17, 0.71]	[−0.47, 0.13]

*Note.* Significant regression coefficients (*p*<.05) are in boldface. By convention, y-standardized regression coefficients of dummy coded variables of the sizes 0.2, 0.5, and 0.8 indicate small, medium, and large effect sizes, respectively. MIMIC  =  multiple indicators multiple causes; DIF  =  differential item functioning; PET  =  positron emission tomography (0 =  no PET, 1 =  PET); Female (0 =  male, 1 =  female); OAV  =  Altered state of consciousness rating scale version (0 =  OAV, 1 =  5D-ASC); Psilocybin (0 =  1.5–1.7 mg/kg MDMA or 6–12 µg ⋅ kg^−1^⋅ min^−1^ ketamine, 1 = 115–315 µg/kg psilocybin); Ketamine (0 = 1.5–1.7 mg/kg MDMA or 115–315 µg/kg psilocybin, 1 = 6–12 µg ⋅ kg^−1^ ⋅ min^−1^ ketamine).

To assure that the parameters of the final model were estimated with sufficient accuracy and that statistical power was high enough to detect significant effects, we performed a Monte Carlo analysis in M*plus* as described by Muthén and Muthén [88]. The parameter values from the final model were used as the population parameter values, the sample size was set to 591, and the model estimation was repeated 10,000 times. The Monte Carlo analysis demonstrated that model parameters and their standard errors were relatively stable and powerful. Specifically, all parameter estimates and their standard errors had bias less than 5%, coverage of all parameter estimates was within the recommended range of 0.91–0.98, and power was higher than 0.8 for all parameter estimates, except for two factor covariances of small effect sizes.

### MIMIC Modeling

The no-DIF MIMIC model showed only slightly reduced global model fit relative to the final CFA model (see Table 2). This suggests that the associations between the covariates and the items were mostly well explained by the indirect effects going through the latent factors. However, by applying the full baseline designated anchor approach to DIF detection, as outlined in the method section, six D-F items were identified (item # 18, 25, 27, 30, 32, and 33; see Table S3 for the meaning of these items). The estimation of a MIMIC model that included direct effects from each covariate to each D–F item (5×6 = 30 direct effects) revealed that nine direct effects were statistically significant at *p*<0.05 and of at least small to moderate effect size (y-standardized regression coefficient >0.2). The final MIMIC model, which accounted for DIF by allowing these 9 direct effects to be freely estimated, fitted significantly better than the no-DIF model (see Table 2), and showed reasonably good global model fit. As with the final CFA model, we performed a Monte Carlo analysis to assure that the parameters of the MIMIC model with DIF adjustment were estimated with sufficient power and accuracy. The analysis confirmed that the parameter estimates and their standard errors were relatively stable and powerful.

From the nine direct effects, six effects (those on item # 18, 25, 27, 30, 32, and 33) were due to measurement non-invariance between the MDMA and ketamine groups. Measurement non-invariance between males and females, between the OAV and 5D-ASC questionnaires, and between the MDMA and psilocybin groups was each accounted for by one direct effect (those on item # 18, 30, and 25, respectively). Whereas the direct effects of the two drug contrasts were well explainable by specific effects of psilocybin and ketamine, the direct effects of the gender and questionnaire version covariates were more difficult to interpret. However, since all estimated direct effects were of only small to moderate effect sizes (all y-standardized regression coefficients were between 0.2 and 0.5), these effects must be interpreted cautiously. Furthermore, because the effects of the covariates on the latent factors in the final MIMIC model with DIF adjustment were not substantially different from those of the no-DIF MIMIC model, the impact of the nine direct effects on the estimated group differences in latent factor means can be considered low. In fact, none of these estimated group differences changed statistical significance as a consequence of controlling for DIF.

The effects of the grouping variables on the latent factors in the DIF-adjusted final MIMIC model are shown in Table 4. Compared to MDMA, psilocybin had the most pronounced effect on scales measuring visual alterations (ie, elementary and complex imagery, audio-visual synesthesiae, and changed meaning of percepts), but also facilitated insights and spiritual experiences and slightly increased anxiety. Ketamine, on the other hand, most strongly reduced blissfulness, increased disembodiment, and impaired control and cognition. Although the effects were less pronounced than those of psilocybin, ketamine also induced visual alterations, most notably elementary imagery, and facilitated spiritual experiences. Furthermore, ketamine slightly increased anxiety compared to MDMA. Averaged over all drugs, females reported more impairment in control and cognition and slightly more/stronger experiences of disembodiment and unity than males. Relative to the 5D-ABC, the OAV questionnaire measured increased changed meaning of percepts, insightfulness, blissful state, and spiritual experiences. Although the different questionnaire lengths and the way items were embedded might have contributed to these differences, it is more plausible that the questionnaire effects were confounded by different drug doses. The average psilocybin doses administered in experiments using the OAV and 5D-ABC were 212 and 251 µg/kg, respectively, whereas the average doses of ketamine were 6 and 12 µg · kg^−1^ · min^−1^, respectively. When drug sessions involved PET measurements, subjects generally experienced stronger subjective drug effects. All scale scores were increased except for the audio-visual synesthesiae and the blissful state scales. The most pronounced effects were observed with respect to visual alterations and disembodiment. The effects of the PET setting might be explained in part by the fact that subjects had more time to concentrate on their experiences when drug sessions took place at the PET center. Specifically, subjects did not have to perform tasks during PET measurements, they were mostly lying in a comfortable horizontal position, and they could have their eyes closed most of the time. However, similar to the questionnaire variable, the setting variable might have been confounded by the effects of different drug doses. The average psilocybin doses administered at the PET center and at the laboratory were 219 and 254 µg/kg, respectively, whereas the average doses of ketamine were 0.79 and 0.87 µg · kg^−1^ · min^−1^, respectively. Although we have controlled the effects of different drug doses in a separate MIMIC model in which we included more dummy variables for different drug groups (ie, dummy variables for low dose psilocybin, low medium dose psilocybin, high medium dose psilocybin, high dose psilocybin, medium dose ketamine, and high dose ketamine with MDMA as the reference group), we decided to provide the results of this analysis as supplementary material only (see Table S7), because a Monte Carlo analysis indicated that the complexity of this model was too high for the size of our sample. Nevertheless, the MIMIC model that included these dose predictors suggested the effects of the PET setting were only slightly confounded by different drug doses. Although the effect sizes were slightly reduced, none of the effects of the setting variable changed statistical significance.

**Table 5:** Reliabilities.

Scale	Items	Cronbach's α	Revelle's β	ρ_SEM_	McDonald's ω_h_	McDonald's ω_tot_
**Original scales**						
Altered state of consciousness	66	.96 [.96, .97]	.61		.65	.97
Oceanic boundlessness	27	.95 [.94, .96]	.71		.74	.96
Dread of ego dissolution	21	.93 [.92, .94]	.74		.74	.94
Visionary restructuralization	18	.91 [.90, .92]	.73		.70	.93
**New scales**						
Experience of unity	5	.88 [.87, .90]	.86	.88 [.87, .90]		
Spiritual experience	3	.77 [.74, .81]	.73	.78 [.73, .83]		
Blissful state	3	.82 [.79, .84]	.79	.82 [.79, .85]		
Insightfulness	3	.73 [.69, .77]	.69	.74 [.69, .79]		
Disembodiment	3	.82 [.80, .85]	.77	.82 [.79, .86]		
Impaired control and cognition	7	.85 [.84, .87]	.80	.86 [.83, .88]		
Anxiety	6	.89 [.88, .90]	.83	.89 [.87, .92]		
Complex imagery	3	.80 [.77, .83]	.77	.80 [.77, .83]		
Elementary imagery	3	.84 [.81, .86]	.73	.86 [.83, .88]		
Audio-visual synesthesiae	3	.91 [.89, .92]	.89	.91 [.89, .93]		
Changed meaning of percepts	3	.79 [.77, .82]	.75	.80 [.76, .84]		

*Note.* Numbers in brackets indicate 95% confidence intervals.

### Reliability Assessment

The results of the reliability assessment of the original and new OAV scales are shown in Table 5. Because the original scales were demonstrated to be multidimensional in the CFA, it was expected that Cronbach's α would be a biased reliability index for these scales. Indeed, a comparison of α with alternative indexes of reliability revealed that α grossly overestimated reliability, when reliability is defined as the proportion of variance in a scale that is due to *one* common factor (McDonald's ω_h_) and slightly underestimated reliability, when reliability is defined as the proportion of variance due to *all* common factors (McDonald's ω_tot_). However, even though variance explained by group factors (ie, factors that are related to a subset of items within a scale) contributed considerably to the very high α coefficients in the original scales, it should be noted that these scales showed relatively large general factor saturations. Specifically, Revelle's β and McDonald's ω_h_ were larger than 0.7 for the OBN, DED and VRS scales and still exceeded 0.6 for the total scale. Thus, although the original scales are not unidimensional, the general factors (ie, factors that are common to all items in a scale) clearly dominated these scales, because they explained more than 70% of the variance in the OBN, DED and VRS scales and more than 60% in the total scale (ie, G-ASC). Because these values exceeded the recommended minimum threshold of Revelle [68], who suggested that the amount of variance explained by the general factor should be at least 50%, and in case of the OBN, DED, and VRS scales even exceeded the more stringent recommendations of Rossiter [89], who suggested aiming for a coefficient β of 0.7, the calculation of sum scores from these scales, including the ASC scale that includes all 66 items, could be justified.

**Table 6:** Correlations between the OAV and the Adjective Mood Rating Scale (EWL60S).

Scale	Efficiency-activation	Concentration	Inactivation	Tiredness	Dazed state	Extroversion	Introversion	Self-confidence	Heightened mood	Emotional excitation	Sensitivity	Aggression-anger	Apprehension-anxiety	Depressiveness	Dreaminess
**Original scales**															
Altered state of consciousness	0.1	−0.14	0.02	−0.05	0.09	−0.04	0.09	−0.03	0.10	0.28***	0.29***	0.01	0.14	0.07	0.24**
Oceanic boundlessness	0.18*	0.01	−0.05	−0.12	0.02	0.06	0.03	0.14	0.23**	0.23**	0.23**	−0.02	0.07	0.01	0.23**
Dread of ego dissolution	−0.01	−0.24**	0.09	0.07	0.10	−0.16*	0.16*	−0.27***	−0.16*	0.27***	0.23**	0.09	0.27***	0.19*	0.10
Visionary restructuralization	0.03	−0.15*	0.03	−0.05	0.12	−0.02	0.05	0.03	0.14	0.18*	0.25***	−0.04	0.00	−0.03	0.27***
**New scales**															
Experience of unity	0.13	0.00	−0.03	−0.13	0.01	0.04	0.04	0.11	0.21**	0.21**	0.21**	−0.04	0.08	0.05	0.28***
Spiritual experience	0.14	0.05	−0.01	−0.08	−0.06	−0.03	0.00	0.09	0.10	0.14	0.21**	0.01	0.09	0.01	0.10
Blissful state	0.19*	0.11	−0.13	−0.12	−0.08	0.18*	−0.04	0.27***	0.39***	0.12	0.17*	−0.01	−0.04	−0.08	0.18*
Insightfulness	0.19*	−0.04	−0.06	−0.09	−0.04	0.02	−0.05	0.09	0.14	0.17*	0.17*	−0.02	0.04	0.00	0.21**
Disembodiment	0.13	−0.04	−0.04	−0.07	0.06	−0.04	0.03	−0.08	0.04	0.18*	0.10	−0.05	0.06	0.02	0.11
Impaired control and cognition	−0.01	−0.24**	0.09	0.03	0.17*	−0.12	0.17*	−0.15*	0.00	0.21**	0.21**	0.02	0.17*	0.08	0.21**
Anxiety	−0.03	−0.19*	0.09	0.11	0.02	−0.15*	0.13	−0.29***	−0.25**	0.24**	0.19*	0.15*	0.32***	0.25***	−0.03
Complex imagery	0.05	−0.11	0.04	−0.01	0.11	0.02	−0.03	0.11	0.12	0.13	0.17*	−0.03	−0.04	−0.06	0.28***
Elementary imagery	−0.10	−0.18*	0.15	0.04	0.17*	−0.12	0.14	−0.02	0.09	0.09	0.17*	−0.01	0.01	−0.03	0.15*
Audio-visual synesthesiae	0.02	0.07	0.02	−0.03	0.06	0.06	0.12	0.09	0.17*	0.11	0.32***	0.07	0.03	0.06	0.17*
Changed meaning of percepts	0.05	−0.24**	−0.04	−0.14	0.04	−0.04	0.00	−0.04	0.05	0.21**	0.16*	−0.12	0.01	−0.04	0.20**

*Note.* * *p*<.05. ** *p*<.01. *** *p*<.001.

When applied to the new OAV scales, Cronbach's α was a less biased estimator of scale reliability. This was expected, as the new OAV scales had been shown to be unidimensional in the CFA. However, because α underestimates reliability when the items of a scale are not tau-equivalent (ie, have unequal factor loadings) and because the assumption of tau-equivalence was not met by most of the new OAV scales, ρ_SEM_ was slightly higher than α in these scales. When estimated by ρ_SEM_, most of the new OAV scales showed good reliabilities. Only two of the 11 scales (insightfulness and spiritual experience) had reliabilities smaller than 0.8. However, both of these scales consisted of only three items and their reliabilities were still above 0.7, which indicates modest reliability [90]. By comparing ρ_SEM_ of the new scales with ω_h_ and ω_tot_ of the old scales it can be seen that, although the new OAV scales have lower reliabilities than the old OAV scales when reliability is defined as the amount of variance in a scale that is due to all common factors, they contain a larger proportion of variance attributable to one common factor. Hence, the new scales are more homogeneous than the old scales. This was also confirmed by the values of coefficient β, which generally were higher for the new scales than for the old OAV scales.

### Validity Assessment

Pearson correlations between the OAV scales and the 15 subscales of the EWL-60-S computed from the raw sum scores are presented in Table 6. The directions and sizes of the correlations between OAV and EWL-60-S subscales covering similar and dissimilar content supported the convergent and discriminant validities of the new OAV scales. For instance, the new OAV anxiety scale correlated highest with the apprehension-anxiety scale of the EWL-60-S, impaired control and cognition correlated highest with concentration, audio-visual synesthesiae correlated highest with sensitivity, complex imagery correlated highest with dreaminess, and blissful state correlated highest with heightened mood. Compared to the old OAV scales, the new OAV scales tended to correlate higher with scales measuring similar experiences. For example, although the OBN scale correlated highest with the EWL-60-S subscale that was hypothesized to cover the most similar content (ie, the heightened mood scale), this correlation (*r* = .27) was considerably lower than the correlation between the more specific blissful state scale and the heightened mood scale (*r* = .37).

Pearson correlations between the OAV and the STAI-S scales showed that the STAI-S total scale was significantly associated with DED (*r* = .59, *p*<.001), anxiety (*r* = .54, *p*<.001), and impaired control and cognition (*r* = .45, *p*<.001). The STAI-S anxiety present scale correlated significantly with DED (*r* = .60, *p*<.001), G-ASC (*r* = .38, *p*<.001), impaired control and cognition (*r* = .52, *p*<.001), anxiety (*r* = .51, *p*<.001), and changed meaning of percepts (*r* = .33, *p* = .012), whereas the STAI-S anxiety absent scale correlated significantly with DED (*r* = .45, *p*<.001), anxiety (*r* = .45, *p*<.001), blissful state (*r* = −.44, *p*<.001), and impaired control and cognition (*r* = .30, *p* = .024). Although these correlations further support the construct validities of the OAV scales, it should be noted that these correlations were calculated on the basis of a relatively small sample of 56 experimental sessions, primarily involving MDMA administration, which is known to rarely induce anxiety or even has anxiolytic effects [91].

**Figure 2:**
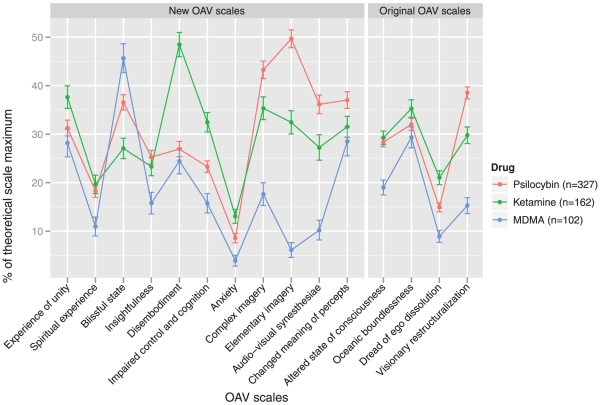
Known-group validities of the original and new OAV scales. Error bars represent standard errors.

Figure 2 displays the mean scores of the new and original OAV scales in the three different drug groups. As can be seen from the plot, the new OAV scales differentiated well among the three drug groups and provided considerably more information on the specific effects of MDMA, ketamine, and psilocybin than the original scales.

## Discussion

This study examined the factorial structure of the OAV questionnaire in a sample of drug induced ASC by using SEM methodology. The results of this study do not support the three dimensional structure originally proposed by the authors of the OAV [5], [7]. The original model provided a poor fit to the data not only when cross-loadings and residual correlations were fixed to zero (simple structure CFA), but also when cross-loadings were freely estimated and residuals of items with similar wording were allowed to freely co-vary (ESEM with method effects) or when an additional general factor was specified (bifactor model).

Although none of the three originally hypothesized OAV factors met criteria of unidimensionality, the results of this study suggest that the VRS factor is the biggest source of misfit. The VRS factor provided not only the worst fit to the data when the three factors were tested separately by one-factor CFAs, it also contained the highest number of items having “wrong” salient factor loadings in the ESEM (six of the seven mis-assigned items were VRS items) and had the lowest general factor saturation. The finding that VRS is the most heterogeneous factor is in agreement with both of the two other studies that have re-examined the factorial structure of the OAV after its first publication. Habermeyer (unpublished MD thesis with partial publication in [12]), who conducted a principal component analysis with varimax-rotation on a sample of 93 endogenous psychotic patients who completed the OAV by referring to their most recent acute psychotic episode, found that 10 of the 18 VRS items loaded highest on the OBN factor. Similarly, in a study of Bodmer [24], in which an EFA with target rotation was conducted using measurements of 135 experimentally induced ASC, 13 VRS items loaded highest on the OBN factor. The VRS items that were wrongly assigned to the OBN factor in the studies of Habermeyer and Bodmer [24], as well as in the present study, are highly congruent. That is, in all three studies, these items describe experiences of changed meaning of percepts, facilitated recollection, and insightfulness. Additionally, in the studies of Habermeyer and Bodmer [24], the wrongly assigned VRS items included items measuring complex imagery. It should be noted that − except for changed meaning of percepts − these facets were not part of the original conceptualization of the VRS dimension (ie, in the original APZ questionnaire), but were introduced during construction of the OAV. Because analyses of the APZ had indicated that the VRS dimension describes not only changes in visual perceptions and their associated meanings, but also a general increase in the perception of internally produced stimuli, Bodmer [5] hypothesized that the VRS dimension could be conceptually extended by incorporating items measuring an increase of imaginations, associations, and memory retrieval. Bodmer's re-conceptualization of the VRS dimension was mainly driven by theoretical considerations of Leuner [22], [23], who had speculated that hallucinogenic drugs elicit visual hallucinations by intensifying internal imagery such that the distinction between internally produced imaginary images and external perceptions becomes blurred. However, given that three studies, including the present study − which has a much larger sample size than the original validation study − have not supported this hypothesis, it appears now that the re-conceptualization of VRS has worsened rather than improved its psychometric properties.

Although reducing the VRS dimension to a set of items tapping only visual alterations would markedly increase its homogeneity, our results indicate that such a construct would still be difficult to separate from the OBN dimension on a high level of the construct hierarchy – especially when potential method effects of similarly worded VRS items are taken into account. Whereas in the three-factorial ESEM without method effects, VRS emerged as a separate factor, it completely merged with the OBN factor when method effects were accounted for by specifying correlated errors. Similarly, the ICLUST algorithm, which seems to be less sensitive to method effects than EFA [69], combined the OBN and VRS factors to one cluster. This suggests that the VRS factor – at least in part – could be an artifact of method effects. Unfortunately, previous OAV validation studies did not consider this possibility, because they exclusively relied on EFA, which cannot account for method effects [70].

Before discussing the issue of what would be the most appropriate number of factors to extract from the OAV, it should be noted that psychological constructs have a hierarchical structure such that different constructs have different levels of conceptual breadth. Hence, the number of factors that can be proposed and assessed is infinite [29]. The appropriate number of factors to extract depends on the appropriate conceptual breadth of a factor, which, in turn, depends on its specific use. For instance, factors on a high level of the construct hierarchy (ie, broad constructs) are best suited to predict heterogeneous/complex criteria, whereas narrow-band factors are most efficacious in predicting a specific criterion [29]. The authors of the OAV decided to extract factors only on a high level of the construct hierarchy because they were primarily interested in the so called etiology-independent dimensions [1], [5]. However, even if only higher order factors are considered, we have not found evidence for a parsimonious fit of a three-factorial solution. The ICLUST procedure indicated that only two factors account for the variance between OAV items on a high level of the construct hierarchy. Whereas one of these two factors was equal to the original DED factor, the other consisted of OBN and VRS items. This suggests that, on a high level, the OAV items are best divided on the basis of whether they describe pleasant (OBN and VRS) or unpleasant (DED) experiences. Revelle's VSS criterion, as well as indexes of general factor saturation, such as Revelle's β and McDonald's ω_H_, indicated that the OAV items could be combined on an even higher level of the construct hierarchy to form a total scale. This finding is in agreement with the originally proposed general factor G-ASC, which is supposed to be a general measure of the alteration in consciousness [5]. According to ω_H_, the general factor accounted for as much as 65% of the common variance between all 66 items of the total scale. Thus, although the total scale is multidimensional and therefore forms ambiguous correlations with other psychological constructs, the general factor saturation is high enough to justify its use for the prediction of complex criteria (cf. [68], [89]). The same is true for the OBN, DED and VRS and the “pleasant” and “unpleasant” scales, which also showed strong general factor saturations despite clear rejection of unidimensionality by CFA.

Although the authors of the OAV have considered lower order scales as unreliable and unstable and therefore refrained from their extraction, this study has demonstrated that a number of lower order scales can be constructed that are not only reliable, but also stable (measurement invariant) and valid. Specifically, by using ICLUST, CFA and MIMIC, we constructed and evaluated 11 new OAV scales formed on the basis of 42 items. The new OAV scales were demonstrated to have many advantages over the old OAV scales. Most importantly, the new scales met criteria of unidimensionality and therefore are more homogeneous than the old scales. Unlike the old scales, the new scales provided a reasonably good fit to the data when modeled as congeneric factors in a simple structure CFA. This is important, because a well fitting CFA model is a prerequisite for further analyses within the SEM-framework. For instance, testing measurement invariance by MIMIC or multiple group CFAs and directly estimating reliabilities and disattenuated correlations with other constructs is not possible without a well fitting basic measurement model [70]. By using a MIMIC model with five binary predictors, the new OAV factors were demonstrated to be highly measurement invariant across three drug groups, two settings, two questionnaire versions and sexes. Although a small number of items showed DIF, especially when comparing the MDMA and ketamine groups, the impact of DIF on the comparisons of latent factor means was small. This is important for the use of these scales in applied research, because it suggests that group mean differences in these scales are hardly confounded by structural differences when calculated on the basis of raw sum scores and by using methods that cannot account for DIF, such as ANOVA. Although the new scales, due to their lower item number, were less reliable than the old scales when reliability is defined as the proportion of variance that is due to all common factors present in a scale, they still showed relatively high reliabilities. Nine scales had reliabilities beyond 0.8 and two scales had reliabilities between 0.7 and 0.8. Because reliability requirements are weaker when scales are used predominantly to compare groups and not for making decisions about individuals (as it is the case with the new OAV scales), reliability indexes of this size can be considered adequate. In fact, it has been argued that increasing reliabilities much beyond 0.8 in basic research is not worth the effort, because measurement error attenuates correlations very little at this level [90]. Indeed, the lower reliabilities of the new OAV scales did not lead to lower correlations with other psychological constructs, such as the subscales of the EWL-60-S. Even though these correlations were based on raw scores, ie were not corrected for measurement error, the new OAV scales tended to correlate more strongly than the old OAV scales. This suggests that the lower reliabilities of new OAV scales were more than compensated by their higher homogeneities. The new scales were also shown to have good convergent and discriminant validities and to differentiate well among the subjective effects of psilocybin, ketamine and MDMA. For example, in the MIMIC model, 10 of the 11 new factors were significantly affected by at least one drug contrast variable. The effects of the drug contrast variables supported the known group validities of the new OAV scales, because the magnitude and direction of the effects were well in line with what is known about these drugs from the scientific literature. Overall, the new OAV scales differentiated better among the three drug groups than the old scales. For example, the very strong effects of MDMA on blissful state and of ketamine on disembodiment would not have been detected by using the original OAV scales alone, because these experiences would have been mixed up with other experiences measured by the OBN scale.

The interpretation of our results with regard to Dittrich's original hypothesis (ie, ASC – independent of their means of induction - can be parsimoniously described by the three oblique primary dimensions OBN, DED, and VRS and the secondary dimension G-ASC) is complicated by the fact that we have analyzed an item set that has been pre-selected to be in accordance with this hypothesis. Unlike the items of the APZ, the items of the OAV were selected and worded to maximally load on one of the three hypothesized primary dimensions [5]. Consequently, the factorial structure of the OAV is most likely reflecting this item selection and cannot provide independent evidence for the validity of Dittrich's hypothesis. Unfortunately, as mentioned in the introduction, Dittrich's factorial structure of ASC may not only be specific to the set of items he selected, it may also be dependent on the data analyzing methods he used. Given these rather severe limitations of Dittrich's original investigations and given that the present study has not confirmed that a three-factorial solution provides a parsimonious fit to the data on a high level of the construct hierarchy, even though the analysis was based on a pre-selected set of items, it seems highly premature to postulate three major dimensions of ASC, let alone to call them etiology-independent.

### Limitations

Because the sample of the present study was too small to split it in two halves and to perform exploratory and confirmatory analyses on separate data sets, we have not cross-validated our results. It is therefore possible that we have capitalized on chance at least to some degree. Furthermore, measurement invariance and population heterogeneity of the new OAV scales were only examined by MIMIC modeling and not by multiple-group CFA. This means that we were only able to test the invariance of indicator intercepts and factor means, and that all other measurement and structural parameters (ie, factor loadings, error variances/covariances, factor variances/covariances) were assumed to be equal across the levels of the covariates. Studies that use multiple-groups CFA are clearly needed to further establish measurement invariance of the new OAV scales. The invariance of measurement and structural parameters should also be investigated across additional groups of drugs, dosages, ASC induction methods, settings, and languages.

Although the newest version of Dittrich's ASC rating scales (ie, the 5D-ASC) contains 94 items, this study has only analyzed the 66 items that it shares with the second newest version (ie, the OAV). Future studies must clarify whether the common variance between the 28 items that are unique to the 5D-ASC is sufficiently well explained by the two hypothesized factors “vigilance reduction” (VIR) and “auditory alterations” (AUA). Since we have shown that the OBN, DED and VRS scales can be split into many reliable and valid subscales, it is conceivable that the same could be done with the VIR and AUA scales.

### Conclusions and Recommendations

The present study confirmed that the general factor (G-ASC) accounts for most of the common variance among OAV items. However, our results only partially supported the hypothesized structure of group factors. Most importantly, we demonstrated that the OBN, DED, and VRS scales are multidimensional constructs that can be split into many reliable and valid subscales. Although the use of the OBN, DED, and VRS scales – due to their relatively strong general factor saturations – might be justified for predicting complex criteria, we believe that our newly constructed subscales should be preferred for most applications, because they are only slightly less reliable but much more homogeneous. Hence, they form less ambiguous correlations with other measures, are easier to interpret, and provide important additional information on more specific experiences of ASC. We especially caution against the use of the VRS factor in its current form, because a relatively large number of its items repeatedly loaded higher on the OBN than on the VRN factor and because its emergence in EFA might be an artifact of method effects.

## Supporting Information

Figure S1 Hierarchical item clustering tree diagram based on Pearson correlations of uncategorized OAV items.
(0.69 MB PDF)

Figure S2 Hierarchical item clustering tree diagram based on polychoric correlations of categorized OAV items.
(0.63 MB PDF)

Table S1 Distributional characteristics of the uncategorized OAV items.
(0.04 MB PDF)

Table S2 Distributional characteristics of the categorized OAV items.
(0.04 MB PDF)

Table S3 Item assignments in the exploratory structural equation model with three factors, geomin rotation, and no correlated residuals.
(0.05 MB PDF)

Table S4 Item assignments in the exploratory structural equation model with three factors, geomin rotation, and correlated residuals.
(0.05 MB PDF)

Table S5 The optimal number of factors to extract determined by different methods.
(0.05 MB PDF)

Table S6 Loading matrices of exploratory structural equation models with an increasing number of factors.
(0.11 MB PDF)

Table S7 Y-standardized regression coeffcients [and 95% confidence intervals] of MIMIC model with dose predictors and DIF.
(0.05 MB PDF)

Methods S1 (0.05 MB DOC)
