# Supplementary figures and images for: Psychometric Evaluation of the Altered States of Consciousness Rating Scale (OAV)
Source: PLoS One. 2010 Aug 31;5(8):e12412. doi: 10.1371/journal.pone.0012412 (PMC2930851; doi:10.1371/journal.pone.0012412)

# Unpleasant Experiences

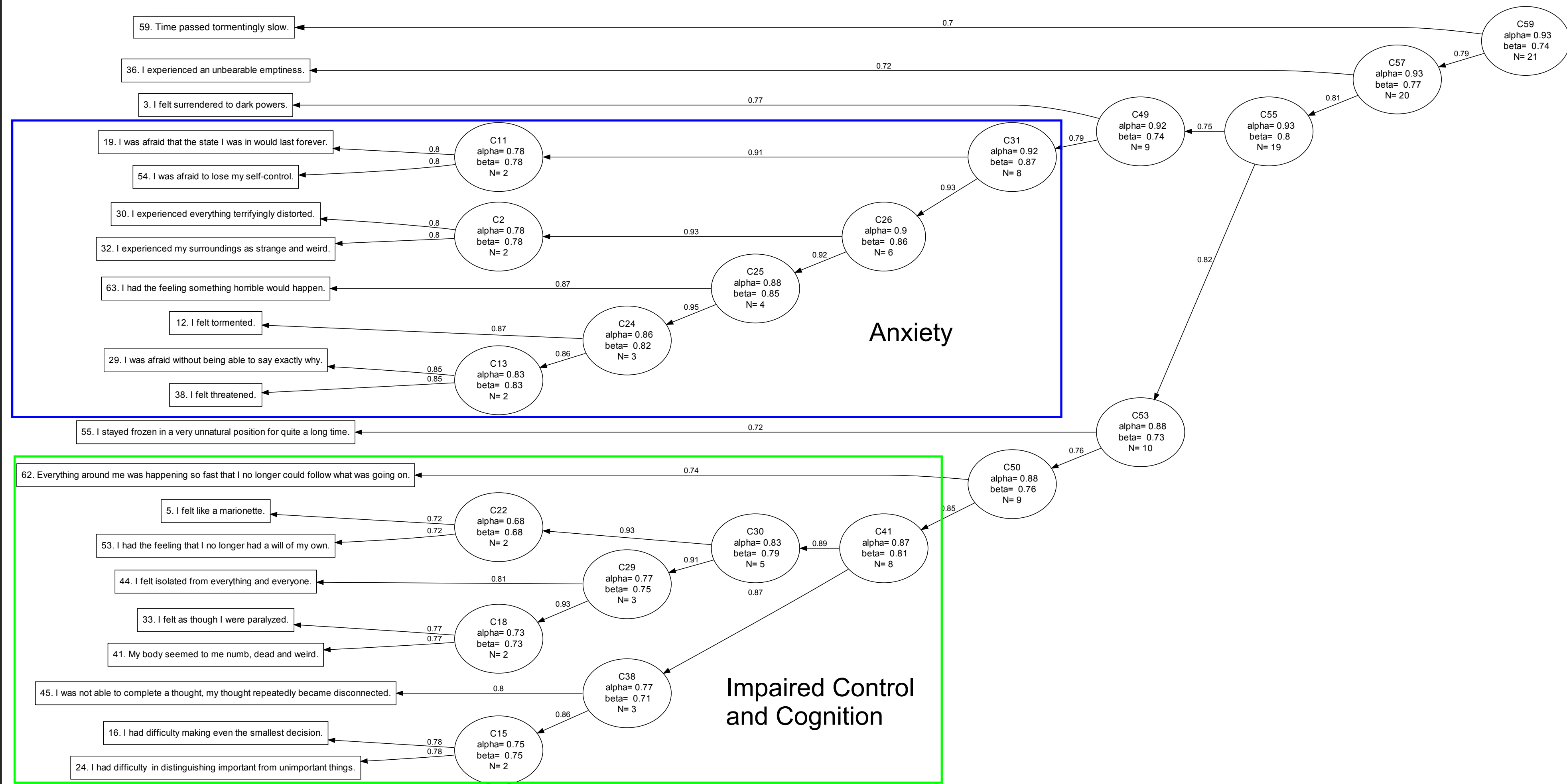

# Pleasant Experiences

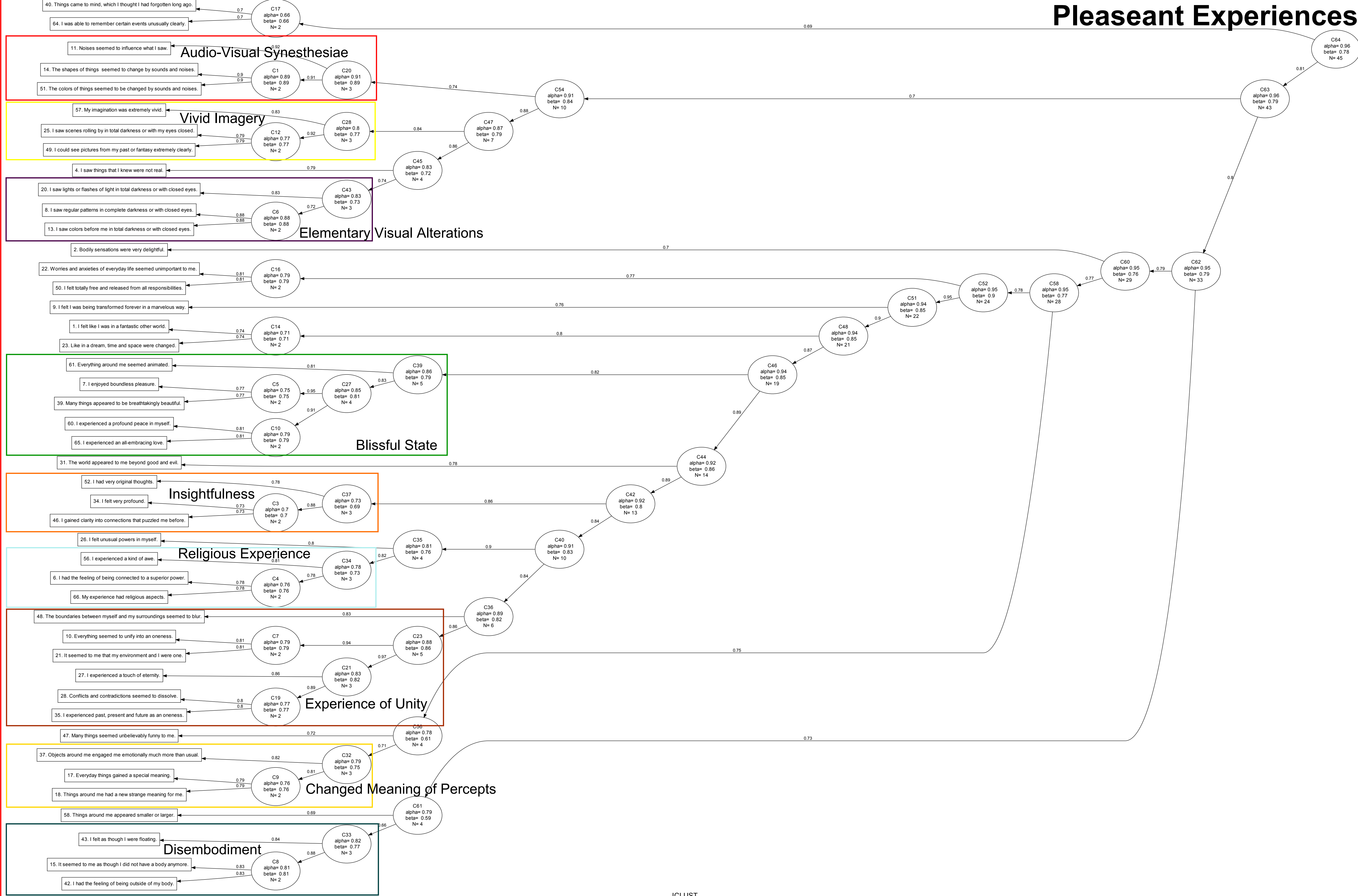

Supplement: Figure S1 — Hierarchical item clustering tree diagram based on Pearson correlations of uncategorized OAV items. (0.69 MB PDF) [file pone.0012412.s001.pdf]

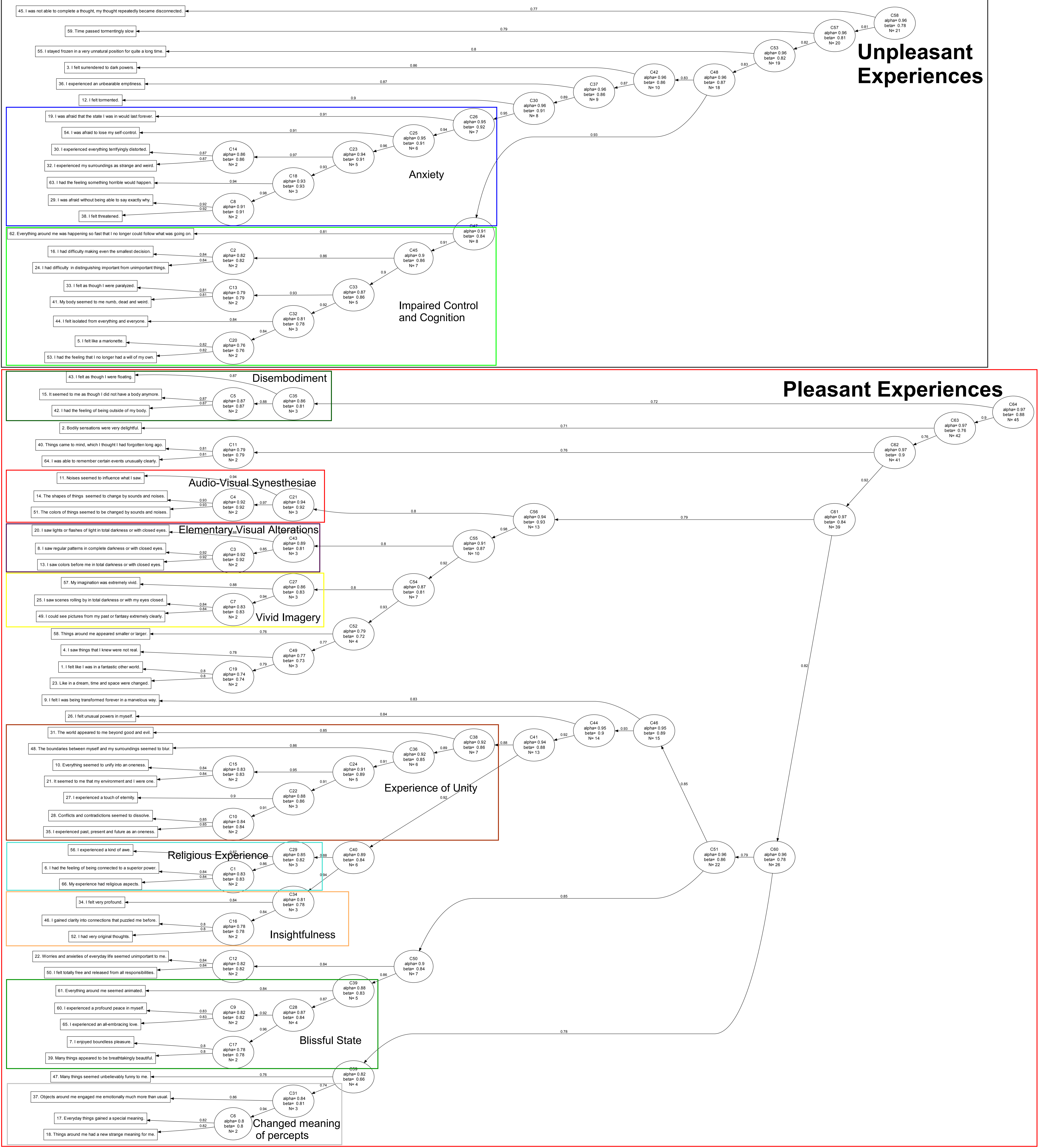

Supplement: Figure S2 — Hierarchical item clustering tree diagram based on polychoric correlations of categorized OAV items. (0.63 MB PDF) [file pone.0012412.s002.pdf]
