# Supplementary material for: Psychometric Evaluation of the Altered States of Consciousness Rating Scale (OAV)
Source: PLoS One. 2010 Aug 31;5(8):e12412. doi: 10.1371/journal.pone.0012412 (PMC2930851; doi:10.1371/journal.pone.0012412)
Supplement: Table S1 — Distributional characteristics of the uncategorized OAV items. (0.04 MB PDF) [file pone.0012412.s003.pdf]

Supplementary Table S1. Distributional characteristics of the uncategorized OAV items.

	mean	sd	median	min	max	skew	kurtosis	% 0 values	% 100 values
**Item**									
1	49.04	34.92	49.00	0	100	0.06	-1.42	9.64	11.00
2	33.90	33.17	21.00	0	100	0.61	-1.03	23.01	4.74
3	5.54	16.51	0.00	0	100	3.90	15.87	71.40	0.68
4	42.06	41.08	32.00	0	100	0.27	-1.64	32.32	15.23
5	15.68	27.02	1.00	0	100	1.91	2.58	49.24	3.21
6	15.76	27.30	0.00	0	100	1.83	2.25	54.31	2.71
7	37.80	32.94	31.00	0	100	0.43	-1.15	18.61	4.91
8	41.77	40.90	28.00	0	100	0.32	-1.61	28.26	14.89
9	20.66	30.50	3.00	0	100	1.43	0.73	43.82	3.55
10	29.77	34.17	16.00	0	100	0.84	-0.72	34.35	5.25
11	30.83	37.06	10.00	0	100	0.80	-0.97	37.73	7.95
12	8.78	20.65	0.00	0	100	2.90	8.01	62.10	1.69
13	43.41	41.07	35.00	0	100	0.25	-1.64	28.43	17.26
14	29.45	36.96	7.00	0	100	0.87	-0.88	39.09	7.78
15	34.61	36.23	21.00	0	100	0.62	-1.12	29.44	8.29
16	25.35	31.02	12.00	0	100	1.12	0.02	32.15	4.23
17	37.14	34.79	28.00	0	100	0.49	-1.16	23.86	7.78
18	32.92	36.47	17.00	0	100	0.69	-1.08	32.99	7.45
19	10.45	24.12	0.00	0	100	2.60	5.70	61.25	2.37
20	27.13	36.07	4.00	0	100	1.00	-0.60	42.81	8.29
21	36.74	34.40	27.00	0	100	0.47	-1.21	23.35	5.41
22	45.99	38.47	47.00	0	100	0.13	-1.57	19.97	13.87
23	64.20	37.07	78.00	0	100	-0.56	-1.25	7.95	27.58
24	36.94	36.49	25.00	0	100	0.55	-1.19	25.04	10.83
25	33.59	40.26	8.00	0	100	0.68	-1.26	39.59	12.69
26	22.13	30.92	4.00	0	100	1.32	0.46	41.62	4.40
27	31.69	36.03	14.00	0	100	0.74	-1.00	34.18	6.43
28	30.21	36.61	10.00	0	100	0.85	-0.86	39.09	7.28
29	9.45	22.47	0.00	0	100	2.85	7.40	64.47	2.71
30	10.82	23.32	0.00	0	100	2.57	5.82	58.21	2.37
31	29.16	36.77	7.00	0	100	0.88	-0.83	39.59	8.63
32	11.65	23.57	0.00	0	100	2.38	4.73	55.50	1.35
33	20.86	29.62	5.00	0	100	1.41	0.72	38.92	2.54
34	28.91	33.09	15.00	0	100	0.88	-0.61	32.99	5.25
35	34.01	37.69	14.00	0	100	0.62	-1.24	33.33	8.29
36	5.92	17.01	0.00	0	100	4.02	16.61	65.14	1.02
37	32.11	36.10	15.00	0	100	0.72	-1.03	33.33	6.94
38	6.66	18.29	0.00	0	100	3.57	12.83	65.31	1.02
39	38.65	36.53	30.00	0	100	0.42	-1.34	25.21	8.63
40	17.86	29.71	1.00	0	100	1.62	1.26	48.90	2.88
41	22.08	31.53	4.00	0	100	1.38	0.55	39.26	3.55
42	26.39	34.71	7.00	0	100	1.07	-0.39	39.26	5.75
43	36.25	36.81	23.00	0	100	0.59	-1.18	26.57	9.31
44	20.09	30.21	3.00	0	100	1.49	0.93	42.64	3.72
45	35.10	35.69	21.00	0	100	0.61	-1.11	24.37	7.95
46	20.75	30.03	2.00	0	100	1.35	0.52	45.01	2.54
47	35.09	36.34	20.00	0	100	0.58	-1.20	27.58	8.29
48	34.10	35.52	21.00	0	100	0.65	-1.04	28.43	7.95
49	29.84	36.20	10.00	0	100	0.83	-0.89	36.55	6.94
50	45.38	37.73	41.00	0	100	0.19	-1.51	18.10	13.54
51	27.43	36.27	5.00	0	100	0.95	-0.74	43.32	6.26
52	19.65	28.45	3.00	0	100	1.43	0.92	44.16	2.88
53	17.62	29.00	1.00	0	100	1.69	1.59	48.05	2.37
54	16.59	29.30	0.00	0	100	1.84	2.11	50.42	4.40
55	13.06	25.32	0.00	0	100	2.17	3.75	54.99	2.37
56	23.14	31.71	4.00	0	100	1.20	0.08	41.29	3.89
57	46.58	36.56	43.00	0	100	0.10	-1.51	16.58	10.83
58	32.61	37.54	13.00	0	100	0.68	-1.16	37.06	8.46
59	15.02	27.72	0.00	0	100	1.98	2.73	53.30	3.72
60	41.45	36.68	37.00	0	100	0.31	-1.41	20.98	8.80
61	24.93	34.34	4.00	0	100	1.12	-0.30	42.30	5.41
62	14.10	25.46	0.00	0	100	2.01	3.08	51.27	2.03
63	5.05	16.07	0.00	0	100	4.32	19.23	68.19	0.85
64	18.54	28.56	1.00	0	100	1.56	1.27	48.05	3.21
65	27.34	32.54	13.00	0	100	0.97	-0.38	34.69	4.23
66	13.36	26.10	0.00	0	100	2.06	3.14	60.24	2.20
**Summary**									
min	5.05	16.07	0.00	0	100	-0.56	-1.64	7.95	0.68
max	64.20	41.08	78.00	0	100	4.32	19.23	71.40	27.58
mean	26.99	32.03	13.50	0	100	1.25	1.27	39.23	6.38
sd	12.31	6.17	15.61	0	0	1.00	4.44	14.55	4.67
