# Supplementary material for: Psychometric Evaluation of the Altered States of Consciousness Rating Scale (OAV)
Source: PLoS One. 2010 Aug 31;5(8):e12412. doi: 10.1371/journal.pone.0012412 (PMC2930851; doi:10.1371/journal.pone.0012412)
Supplement: Table S2 — Distributional characteristics of the categorized OAV items. (0.04 MB PDF) [file pone.0012412.s004.pdf]

Supplementary Table S2. Distributional characteristics of the categorized OAV items.

	mean	sd	median	min	max	skew	kurtosis	% 1 values	% 5 values
**Items**									
1	3.06	1.25	3.00	1	5	0.17	-1.01	9.64	28.76
2	2.50	1.19	2.00	1	5	0.50	-0.61	7.78	32.15
3	1.39	0.74	1.00	1	5	2.45	7.14	1.02	71.40
4	2.77	1.57	3.00	1	5	0.24	-1.48	12.35	32.32
5	1.83	1.07	2.00	1	5	1.44	1.62	3.21	49.24
6	1.78	1.07	1.00	1	5	1.47	1.60	3.05	54.31
7	2.65	1.20	3.00	1	5	0.38	-0.69	9.64	29.78
8	2.81	1.55	2.00	1	5	0.27	-1.44	9.64	28.26
9	2.00	1.17	2.00	1	5	1.16	0.51	5.41	43.82
10	2.31	1.29	2.00	1	5	0.75	-0.47	7.45	34.35
11	2.37	1.42	2.00	1	5	0.69	-0.87	9.14	37.73
12	1.54	0.86	1.00	1	5	2.00	4.48	1.35	62.10
13	2.84	1.55	3.00	1	5	0.20	-1.46	11.34	28.43
14	2.33	1.41	2.00	1	5	0.74	-0.80	9.64	39.09
15	2.52	1.37	2.00	1	5	0.54	-0.90	9.14	29.44
16	2.21	1.16	2.00	1	5	0.87	0.02	6.77	34.69
17	2.60	1.29	2.00	1	5	0.46	-0.78	9.64	27.07
18	2.45	1.38	2.00	1	5	0.59	-0.90	10.49	32.99
19	1.62	0.98	1.00	1	5	1.91	3.35	3.05	61.25
20	2.22	1.38	2.00	1	5	0.88	-0.52	7.28	42.81
21	2.59	1.26	2.00	1	5	0.44	-0.78	10.83	28.26
22	2.96	1.43	3.00	1	5	0.11	-1.32	15.23	23.35
23	3.65	1.37	4.00	1	5	-0.49	-1.12	7.95	41.62
24	2.62	1.36	2.00	1	5	0.48	-0.93	9.48	28.26
25	2.47	1.55	2.00	1	5	0.61	-1.17	6.77	39.59
26	2.05	1.18	2.00	1	5	1.07	0.33	5.08	41.62
27	2.40	1.35	2.00	1	5	0.63	-0.82	10.83	34.18
28	2.34	1.41	2.00	1	5	0.72	-0.80	7.78	39.09
29	1.54	0.92	1.00	1	5	2.11	4.45	2.20	64.47
30	1.63	0.96	1.00	1	5	1.89	3.53	2.03	58.21
31	2.31	1.40	2.00	1	5	0.77	-0.71	8.12	39.59
32	1.65	0.92	1.00	1	5	1.70	2.89	2.37	55.50
33	2.03	1.11	2.00	1	5	1.06	0.43	4.57	38.92
34	2.31	1.25	2.00	1	5	0.72	-0.46	8.46	32.99
35	2.49	1.42	2.00	1	5	0.54	-1.03	11.00	33.33
36	1.45	0.75	1.00	1	5	2.37	7.24	1.35	65.14
37	2.42	1.37	2.00	1	5	0.62	-0.82	9.48	33.33
38	1.47	0.79	1.00	1	5	2.22	5.90	1.35	65.31
39	2.68	1.37	3.00	1	5	0.35	-1.06	12.01	25.21
40	1.90	1.15	2.00	1	5	1.32	0.92	5.41	48.90
41	2.09	1.22	2.00	1	5	1.15	0.42	4.91	39.26
42	2.22	1.33	2.00	1	5	0.91	-0.34	6.26	39.26
43	2.59	1.37	2.00	1	5	0.51	-0.93	8.80	27.92
44	2.02	1.18	2.00	1	5	1.17	0.55	5.25	42.64
45	2.59	1.32	2.00	1	5	0.50	-0.87	11.00	30.29
46	1.99	1.15	2.00	1	5	1.09	0.39	5.41	45.01
47	2.55	1.35	2.00	1	5	0.51	-0.92	10.83	27.58
48	2.51	1.33	2.00	1	5	0.56	-0.77	7.95	28.43
49	2.33	1.36	2.00	1	5	0.76	-0.66	8.29	36.55
50	2.94	1.40	3.00	1	5	0.19	-1.24	11.68	25.21
51	2.23	1.39	2.00	1	5	0.84	-0.63	8.80	43.32
52	1.96	1.10	2.00	1	5	1.11	0.63	4.23	44.16
53	1.90	1.14	2.00	1	5	1.35	1.08	4.06	48.05
54	1.86	1.15	1.00	1	5	1.45	1.34	3.55	50.42
55	1.71	1.00	1.00	1	5	1.66	2.48	2.71	54.99
56	2.10	1.21	2.00	1	5	0.97	0.02	6.77	41.29
57	2.97	1.35	3.00	1	5	0.11	-1.16	16.24	24.70
58	2.42	1.42	2.00	1	5	0.61	-0.97	10.49	37.06
59	1.79	1.09	1.00	1	5	1.51	1.63	4.40	53.30
60	2.79	1.36	3.00	1	5	0.28	-1.10	13.03	25.21
61	2.17	1.32	2.00	1	5	0.92	-0.36	8.80	42.30
62	1.76	1.00	1.00	1	5	1.49	1.94	3.38	51.27
63	1.41	0.72	1.00	1	5	2.46	7.77	1.02	68.19
64	1.90	1.10	2.00	1	5	1.24	0.85	4.57	48.05
65	2.26	1.24	2.00	1	5	0.79	-0.29	6.09	34.69
66	1.68	1.04	1.00	1	5	1.68	2.30	2.37	60.24
**Summary**									
min	1.39	0.72	1.00	1	5	-0.49	-1.48	1.02	23.35
max	3.65	1.57	4.00	1	5	2.46	7.77	16.24	71.40
mean	2.23	1.23	1.94	1	5	0.95	0.49	7.12	41.00
sd	0.47	0.20	0.65	0	0	0.64	2.20	3.64	12.37

Note. Items were categorizes as follows: 0 = 1, 1-29 = 2, 30-69 = 3, 70-89 = 4, 90-100 = 5.
