# Supplementary material for: Psychometric Evaluation of the Altered States of Consciousness Rating Scale (OAV)
Source: PLoS One. 2010 Aug 31;5(8):e12412. doi: 10.1371/journal.pone.0012412 (PMC2930851; doi:10.1371/journal.pone.0012412)
Supplement: Table S3 — Item assignments in the exploratory structural equation model with three factors, geomin rotation, and no correlated residuals. (0.05 MB PDF) [file pone.0012412.s005.pdf]

Supplementary Table S3. Item assignments in the exploratory structural equaion model with three factors, geomin rotation, and no correlated residuals.

	Item #	Highest loading	Hypothesized Item-factor membership
**Factor 1**			
I experienced an all-embracing love.	65	0.80	OBN
It seemed to me that my environment and I were one.	21	0.79	OBN
I experienced a touch of eternity.	27	0.77	OBN
I experienced a profound peace in myself.	60	0.73	OBN
Conflicts and contradictions seemed to dissolve.	28	0.72	OBN
Everything seemed to unify into an oneness.	10	0.71	OBN
I felt totally free and released from all responsibilities.	50	0.70	OBN
Everything around me seemed animated.	61	0.70	OBN
Worries and anxieties of everyday life seemed unimportant to me.	22	0.64	OBN
I enjoyed boundless pleasure.	7	0.63	OBN
My experience had religious aspects.	66	0.63	OBN
I felt unusual powers in myself.	26	0.61	OBN
I experienced past, present and future as an oneness.	35	0.60	OBN
I had the feeling of being connected to a superior power.	6	0.58	OBN
Many things appeared to be breathtakingly beautiful.	39	0.57	OBN
The world appeared to me beyond good and evil.	31	0.57	OBN
I experienced a kind of awe.	56	0.57	OBN
The boundaries between myself and my surroundings seemed to blur.	48	0.55	OBN
I felt as though I were floating.	43	0.53	OBN
I felt like I was in a fantastic other world.	1	0.52	OBN
I felt I was being transformed forever in a marvelous way.	9	0.51	OBN
I gained clarity into connections that puzzled me before.	46	0.50	OBN
I felt very profound.	34	0.49	OBN
It seemed to me as though I did not have a body anymore.	15	0.49	OBN
I had the feeling of being outside of my body.	42	0.47	OBN
I had very original thoughts.	52	0.46	VRS
Bodily sensations were very delightful.	2	0.45	OBN
Objects around me engaged me emotionally much more than usual.	37	0.42	VRS
Things around me had a new strange meaning for me.	18	0.42	VRS
Like in a dream, time and space were changed.	23	0.38	OBN
Everyday things gained a special meaning.	17	0.35	VRS
Things came to mind, which I thought I had forgotten long ago.	40	0.34	VRS
I was able to remember certain events unusually clearly.	64	0.28	VRS
**Factor 2**			
I felt threatened.	38	0.78	DED
I was afraid without being able to say exactly why.	29	0.76	DED
I was afraid to lose my self-control.	54	0.76	DED
I experienced my surroundings as strange and weird.	32	0.76	DED
I experienced everything terrifyingly distorted.	30	0.75	DED
I had the feeling something horrible would happen.	63	0.74	DED
I felt tormented.	12	0.73	DED
I was afraid that the state I was in would last forever.	19	0.70	DED
I felt as though I were paralyzed.	33	0.64	DED
I felt like a marionette.	5	0.62	DED
I had the feeling that I no longer had a will of my own.	53	0.59	DED
I felt isolated from everything and everyone.	44	0.58	DED
My body seemed to me numb, dead and weird.	41	0.58	DED
I felt surrendered to dark powers.	3	0.56	DED
I had difficulty making even the smallest decision.	16	0.54	DED
I experienced an unbearable emptiness.	36	0.53	DED
Time passed tormentingly slow	59	0.51	DED
I had difficulty in distinguishing important from unimportant things.	24	0.49	DED
Everything around me was happening so fast that I no longer could follow what was going on.	62	0.48	DED
I stayed frozen in a very unnatural position for quite a long time.	55	0.42	DED
I was not able to complete a thought, my thought repeatedly became disconnected.	45	0.38	DED
Things around me appeared smaller or larger.	58	0.32	VRS
**Factor 3**			
I saw colors before me in total darkness or with closed eyes.	13	0.90	VRS
I saw regular patterns in complete darkness or with closed eyes.	8	0.84	VRS
The shapes of things seemed to change by sounds and noises.	14	0.76	VRS
The colors of things seemed to be changed by sounds and noises.	51	0.75	VRS
I saw scenes rolling by in total darkness or with my eyes closed.	25	0.65	VRS
Noises seemed to influence what I saw.	11	0.61	VRS
I saw lights or flashes of light in total darkness or with closed eyes.	20	0.61	VRS
I saw things that I knew were not real.	4	0.57	VRS
I could see pictures from my past or fantasy extremely clearly.	49	0.51	VRS
My imagination was extremely vivid.	57	0.47	VRS
Many things seemed unbelievably funny to me.	47	0.39	VRS

Note. Items were assigned to the factor on which they loaded most strongly. Items not loading most strongly on their hypothesized factor are in red. OBN = oceanic boundlessness; DED = dread of ego dissolution; VRS = visionary restructuralization.
