# Supplementary material for: Psychometric Evaluation of the Altered States of Consciousness Rating Scale (OAV)
Source: PLoS One. 2010 Aug 31;5(8):e12412. doi: 10.1371/journal.pone.0012412 (PMC2930851; doi:10.1371/journal.pone.0012412)
Supplement: Table S4 — Item assignments in the exploratory structural equation model with three factors, geomin rotation, and correlated residuals. (0.05 MB PDF) [file pone.0012412.s006.pdf]

Supplementary Table S4. Item assignments in the exploratory structural equaion model with three factors, geomin rotation, and correlated residuals.

	Item #	Highest loading	Hypothesized Item-factor membership
**Factor 1**			
I experienced an all-embracing love.	65	0.80	OBN
Many things appeared to be breathtakingly beautiful.	39	0.75	OBN
My experience had religious aspects.	66	0.74	OBN
I experienced a touch of eternity.	27	0.73	OBN
Everything seemed to unify into an oneness.	10	0.70	OBN
Conflicts and contradictions seemed to dissolve.	28	0.70	OBN
I had the feeling of being connected to a superior power.	6	0.68	OBN
I felt unusual powers in myself.	26	0.67	OBN
I experienced a profound peace in myself.	60	0.67	OBN
Everything around me seemed animated.	61	0.67	OBN
It seemed to me that my environment and I were one.	21	0.66	OBN
I enjoyed boundless pleasure.	7	0.65	OBN
I experienced a kind of awe.	56	0.64	OBN
I gained clarity into connections that puzzled me before.	46	0.64	OBN
I felt very profound.	34	0.63	OBN
I had very original thoughts.	52	0.58	VRS
I felt totally free and released from all responsibilities.	50	0.57	OBN
I felt like I was in a fantastic other world.	1	0.53	OBN
I felt I was being transformed forever in a marvelous way.	9	0.53	OBN
I experienced past, present and future as an oneness.	35	0.53	OBN
My imagination was extremely vivid.	57	0.52	VRS
Worries and anxieties of everyday life seemed unimportant to me.	22	0.51	OBN
Things came to mind, which I thought I had forgotten long ago.	40	0.51	VRS
Objects around me engaged me emotionally much more than usual.	37	0.46	VRS
The world appeared to me beyond good and evil.	31	0.46	OBN
Bodily sensations were very delightful.	2	0.45	OBN
Noises seemed to influence what I saw.	11	0.45	VRS
I could see pictures from my past or fantasy extremely clearly.	49	0.44	VRS
The colors of things seemed to be changed by sounds and noises.	51	0.44	VRS
I was able to remember certain events unusually clearly.	64	0.42	VRS
The shapes of things seemed to change by sounds and noises.	14	0.39	VRS
Everyday things gained a special meaning.	17	0.38	VRS
I saw scenes rolling by in total darkness or with my eyes closed.	25	0.35	VRS
Many things seemed unbelievably funny to me.	47	0.34	VRS
I saw colors before me in total darkness or with closed eyes.	13	0.34	VRS
I saw regular patterns in complete darkness or with closed eyes.	8	0.29	VRS
I saw lights or flashes of light in total darkness or with closed eyes.	20	0.29	VRS
**Factor 2**			
My body seemed to me numb, dead and weird.	41	0.71	DED
I felt as though I were paralyzed.	33	0.70	DED
I felt isolated from everything and everyone.	44	0.68	DED
I had difficulty in distinguishing important from unimportant things.	24	0.67	DED
I had the feeling that I no longer had a will of my own.	53	0.64	DED
I had difficulty making even the smallest decision.	16	0.63	DED
I felt like a marionette.	5	0.60	DED
I was afraid to lose my self-control.	54	0.58	DED
I was not able to complete a thought, my thought repeatedly became disconnected.	45	0.58	DED
I experienced my surroundings as strange and weird.	32	0.55	DED
I experienced everything terrifyingly distorted.	30	0.54	DED
I had the feeling of being outside of my body.	42	0.51	OBN
Everything around me was happening so fast that I no longer could follow what was going on.	62	0.51	DED
Things around me appeared smaller or larger.	58	0.50	VRS
Like in a dream, time and space were changed.	23	0.49	OBN
It seemed to me as though I did not have a body anymore.	15	0.49	OBN
I stayed frozen in a very unnatural position for quite a long time.	55	0.47	DED
The boundaries between myself and my surroundings seemed to blur.	48	0.45	OBN
Things around me had a new strange meaning for me.	18	0.41	VRS
I felt as though I were floating.	43	0.38	OBN
Time passed tormentingly slow	59	0.38	DED
I saw things that I knew were not real.	4	0.37	VRS
**Factor 3**			
I felt threatened.	38	0.66	DED
I was afraid without being able to say exactly why.	29	0.65	DED
I felt tormented.	12	0.63	DED
I had the feeling something horrible would happen.	63	0.62	DED
I was afraid that the state I was in would last forever.	19	0.52	DED
I felt surrendered to dark powers.	3	0.42	DED
I experienced an unbearable emptiness.	36	0.37	DED

Note. Residual correlations between the items 8, 13, 20, and 25 and between the items 14 and 51 were freely estimated. Items were assigned to the factor on which they loaded most strongly. Items not loading most strongly on their hypothesized factor are in red. OBN = oceanic boundlessness; DED = dread of ego dissolution; VRS = visionary restructuralization.
