# Supplementary material for: Psychometric Evaluation of the Altered States of Consciousness Rating Scale (OAV)
Source: PLoS One. 2010 Aug 31;5(8):e12412. doi: 10.1371/journal.pone.0012412 (PMC2930851; doi:10.1371/journal.pone.0012412)
Supplement: Table S5 — The optimal number of factors to extract determined by different methods. (0.05 MB PDF) [file pone.0012412.s007.pdf]

Supplementary Table S5. The optimal number of factors to extract determined by different methods.

	MDMA<br>*n* = 102	Ketamine<br>*n* = 162	Psilocybin<br>*n* = 327	Combined<br>*n* = 591
Scree test	6	5	5	5
PA with principal components and mean criterion	6	5	5	5
PA with principal components and 95% percentile criterion	6	5	5	5
PA with principal factors and mean criterion	7	5	7	7
PA with principal factors and 95% percentile criterion	7	5	7	7
Minimum average partial test	9	5	7	7
ICLUST procedure with default settings	4	2	2	2
VSS criterion with complexity 1	1	1	1	1
VSS criterion with complexity 2	4	2	2	2
VSS criterion with complexity 3	3	4	3	4

Note. PA = parallel analysis; ICLUST = item cluster analysis; VSS = very simple structure.
