# Supplementary material for: Psychometric Evaluation of the Altered States of Consciousness Rating Scale (OAV)
Source: PLoS One. 2010 Aug 31;5(8):e12412. doi: 10.1371/journal.pone.0012412 (PMC2930851; doi:10.1371/journal.pone.0012412)
Supplement: Table S6 — Loading matrices of exploratory structural equation models with an increasing number of factors. (0.11 MB PDF) [file pone.0012412.s008.pdf]

Supplementary Table S6. Loading matrices of exploratoy structural equation models with an increasing number of factors.

	f1	f2	f3
**f1**			
65. I experienced an all-embracing love.	0.797	-0.268	
21. It seemed to me that my environment and I were one.	0.788		
27. I experienced a touch of eternity.	0.773		
60. I experienced a profound peace in myself.	0.726	-0.269	
28. Conflicts and contradictions seemed to dissolve.	0.723		
10. Everything seemed to unify into an oneness.	0.712		
50. I felt totally free and released from all responsibilities.	0.704	-0.111	
61. Everything around me seemed animated.	0.697		
22. Worries and anxieties of everyday life seemed unimportant to me.	0.635		
7. I enjoyed boundless pleasure.	0.630	-0.199	0.119
66. My experience had religious aspects.	0.627		
26. I felt unusual powers in myself.	0.612		
35. I experienced past, present and future as an oneness.	0.597	0.197	
6. I had the feeling of being connected to a superior power.	0.577		
39. Many things appeared to be breathtakingly beautiful.	0.574	-0.164	0.318
31. The world appeared to me beyond good and evil.	0.571	0.277	
56. I experienced a kind of awe.	0.571		
48. The boundaries between myself and my surroundings seemed to blur.	0.548	0.279	
43. I felt as though I were floating.	0.532	0.199	
1. I felt like I was in a fantastic other world.	0.518		0.206
9. I felt I was being transformed forever in a marvelous way.	0.514	0.181	
46. I gained clarity into connections that puzzled me before.	0.501		
34. I felt very profound.	0.489		0.111
15. It seemed to me as though I did not have a body anymore.	0.485	0.306	
42. I had the feeling of being outside of my body.	0.468	0.342	
52. I had very original thoughts.	0.459		0.168
2. Bodily sensations were very delightful.	0.450		0.106
37. Objects around me engaged me emotionally much more than usual.	0.425		0.109
18. Things around me had a new strange meaning for me.	0.424	0.306	0.107
23. Like in a dream, time and space were changed.	0.379	0.214	0.288
17. Everyday things gained a special meaning.	0.354	0.241	0.188
40. Things came to mind, which I thought I had forgotten long ago.	0.336		0.209
64. I was able to remember certain events unusually clearly.	0.280		0.274
**f2**			
38. I felt threatened.		0.783	
29. I was afraid without being able to say exactly why.		0.762	
32. I experienced my surroundings as strange and weird.		0.759	
54. I was afraid to lose my self-control.		0.759	
30. I experienced everything terrifyingly distorted.		0.749	
63. I had the feeling something horrible would happen.	-0.134	0.741	
12. I felt tormented.	-0.208	0.733	0.114
19. I was afraid that the state I was in would last forever.		0.695	
33. I felt as though I were paralyzed.		0.640	
5. I felt like a marionette.		0.620	
53. I had the feeling that I no longer had a will of my own.	0.233	0.594	
44. I felt isolated from everything and everyone.		0.583	
41. My body seemed to me numb, dead and weird.	0.176	0.579	
3. I felt surrendered to dark powers.		0.559	
16. I had difficulty making even the smallest decision.	0.142	0.536	
36. I experienced an unbearable emptiness.		0.533	
59. Time passed tormentingly slow	-0.112	0.508	0.109
24. I had difficulty in distinguishing important from unimportant things.	0.298	0.491	
62. Everything around me was happening so fast that I no longer could follow what was going on.		0.483	0.135
55. I stayed frozen in a very unnatural position for quite a long time.	0.139	0.424	
45. I was not able to complete a thought, my thought repeatedly became disconnected.		0.384	0.255
58. Things around me appeared smaller or larger.	0.138	0.324	0.231
**f3**			
13. I saw colors before me in total darkness or with closed eyes.	-0.135		0.896
8. I saw regular patterns in complete darkness or with closed eyes.	-0.158		0.842
14. The shapes of things seemed to change by sounds and noises.			0.760
51. The colors of things seemed to be changed by sounds and noises.			0.750
25. I saw scenes rolling by in total darkness or with my eyes closed.			0.648
11. Noises seemed to influence what I saw.	0.152		0.610
20. I saw lights or flashes of light in total darkness or with closed eyes.			0.610
4. I saw things that I knew were not real.		0.155	0.572
49. I could see pictures from my past or fantasy extremely clearly.	0.187		0.515
57. My imagination was extremely vivid.	0.326		0.470
47. Many things seemed unbelievably funny to me.	0.172		0.392

Note. Loadings < 0.1 are not shown. Cross-loadings > half the size of the main factor loadings are in red.

	f1	f2	f3	f4
**f1**				
65. I experienced an all-embracing love.	0.827		-0.109	-0.137
66. My experience had religious aspects.	0.749		0.170	-0.213
27. I experienced a touch of eternity.	0.744			0.137
10. Everything seemed to unify into an oneness.	0.681			0.144
21. It seemed to me that my environment and I were one.	0.673		-0.117	0.264
61. Everything around me seemed animated.	0.673			
28. Conflicts and contradictions seemed to dissolve.	0.669			0.186
6. I had the feeling of being connected to a superior power.	0.657		0.166	
60. I experienced a profound peace in myself.	0.655		-0.258	
26. I felt unusual powers in myself.	0.652		0.104	
56. I experienced a kind of awe.	0.621		0.159	
39. Many things appeared to be breathtakingly beautiful.	0.601	0.304		
46. I gained clarity into connections that puzzled me before.	0.600		0.224	-0.126
7. I enjoyed boundless pleasure.	0.584	0.118	-0.176	
34. I felt very profound.	0.580		0.222	-0.108
50. I felt totally free and released from all responsibilities.	0.544		-0.272	0.319
9. I felt I was being transformed forever in a marvelous way.	0.526		0.186	
52. I had very original thoughts.	0.505	0.160		
22. Worries and anxieties of everyday life seemed unimportant to me.	0.499		-0.160	0.299
35. I experienced past, present and future as an oneness.	0.477			0.375
31. The world appeared to me beyond good and evil.	0.465			0.360
1. I felt like I was in a fantastic other world.	0.425	0.206		0.259
40. Things came to mind, which I thought I had forgotten long ago.	0.423	0.197	0.144	-0.130
37. Objects around me engaged me emotionally much more than usual.	0.409	0.114		
2. Bodily sensations were very delightful.	0.401	0.109		
18. Things around me had a new strange meaning for me.	0.349	0.114	0.149	0.301
64. I was able to remember certain events unusually clearly.	0.299	0.264		
17. Everyday things gained a special meaning.	0.292	0.196	0.110	0.250
**f2**				
13. I saw colors before me in total darkness or with closed eyes.		0.865		
8. I saw regular patterns in complete darkness or with closed eyes.		0.814		
14. The shapes of things seemed to change by sounds and noises.		0.731		
51. The colors of things seemed to be changed by sounds and noises.	0.103	0.721		
25. I saw scenes rolling by in total darkness or with my eyes closed.		0.626		
20. I saw lights or flashes of light in total darkness or with closed eyes.		0.585		
11. Noises seemed to influence what I saw.	0.182	0.583		
4. I saw things that I knew were not real.		0.561		0.162
49. I could see pictures from my past or fantasy extremely clearly.	0.205	0.497		
57. My imagination was extremely vivid.	0.298	0.461		0.129
47. Many things seemed unbelievably funny to me.	0.161	0.389		
**f3**				
38. I felt threatened.			0.808	
29. I was afraid without being able to say exactly why.			0.793	
12. I felt tormented.		0.109	0.779	
63. I had the feeling something horrible would happen.			0.762	
19. I was afraid that the state I was in would last forever.			0.658	0.101
32. I experienced my surroundings as strange and weird.			0.627	0.248
30. I experienced everything terrifyingly distorted.			0.620	0.229
54. I was afraid to lose my self-control.			0.597	0.287
3. I felt surrendered to dark powers.			0.526	
36. I experienced an unbearable emptiness.			0.480	0.124
59. Time passed tormentingly slow		0.110	0.443	0.118
**f4**				
41. My body seemed to me numb, dead and weird.			0.155	0.680
33. I felt as though I were paralyzed.	-0.125		0.269	0.589
24. I had difficulty in distinguishing important from unimportant things.	0.129		0.145	0.570
42. I had the feeling of being outside of my body.	0.289			0.559
15. It seemed to me as though I did not have a body anymore.	0.304			0.551
44. I felt isolated from everything and everyone.			0.241	0.546
53. I had the feeling that I no longer had a will of my own.			0.269	0.540
16. I had difficulty making even the smallest decision.			0.230	0.493
43. I felt as though I were floating.	0.360			0.475
48. The boundaries between myself and my surroundings seemed to blur.	0.398			0.466
5. I felt like a marionette.			0.345	0.442
23. Like in a dream, time and space were changed.	0.240	0.293		0.428
45. I was not able to complete a thought, my thought repeatedly became disconnected.		0.268	0.114	0.413
58. Things around me appeared smaller or larger.		0.237		0.383
55. I stayed frozen in a very unnatural position for quite a long time.			0.209	0.359
62. Everything around me was happening so fast that I no longer could follow what was going on.		0.142	0.288	0.312

	f1	f2	f3	f4	f5
**f1**					
14. The shapes of things seemed to change by sounds and noises.	0.845				-0.154
13. I saw colors before me in total darkness or with closed eyes.	0.826				
51. The colors of things seemed to be changed by sounds and noises.	0.814				-0.113
8. I saw regular patterns in complete darkness or with closed eyes.	0.754	0.101			
11. Noises seemed to influence what I saw.	0.695				-0.190
25. I saw scenes rolling by in total darkness or with my eyes closed.	0.601				
20. I saw lights or flashes of light in total darkness or with closed eyes.	0.567				
4. I saw things that I knew were not real.	0.501	0.100	0.108		0.155
49. I could see pictures from my past or fantasy extremely clearly.	0.482			0.203	
57. My imagination was extremely vivid.	0.380			0.361	0.177
**f2**					
38. I felt threatened.		0.826			
29. I was afraid without being able to say exactly why.		0.804			
12. I felt tormented.	0.107	0.796			
63. I had the feeling something horrible would happen.		0.773			
19. I was afraid that the state I was in would last forever.		0.686			
32. I experienced my surroundings as strange and weird.		0.669	0.200		
30. I experienced everything terrifyingly distorted.		0.668	0.169		
54. I was afraid to lose my self-control.		0.653	0.223		
3. I felt surrendered to dark powers.		0.533			-0.100
36. I experienced an unbearable emptiness.		0.501	0.100		
59. Time passed tormentingly slow		0.482			
62. Everything around me was happening so fast that I no longer could follow what was going on.		0.356	0.243		0.168
**f3**					
41. My body seemed to me numb, dead and weird.		0.204	0.720	-0.192	
15. It seemed to me as though I did not have a body anymore.			0.700		-0.293
42. I had the feeling of being outside of my body.			0.691		-0.251
33. I felt as though I were paralyzed.		0.337	0.566	-0.215	
43. I felt as though I were floating.			0.564	0.207	-0.131
48. The boundaries between myself and my surroundings seemed to blur.			0.527	0.271	
53. I had the feeling that I no longer had a will of my own.		0.328	0.523		
24. I had difficulty in distinguishing important from unimportant things.		0.232	0.516	0.117	0.204
44. I felt isolated from everything and everyone.		0.315	0.500	-0.101	0.120
35. I experienced past, present and future as an oneness.	0.113		0.461	0.337	-0.165
31. The world appeared to me beyond good and evil.			0.428	0.346	-0.138
16. I had difficulty making even the smallest decision.		0.316	0.426		0.203
23. Like in a dream, time and space were changed.	0.238		0.417	0.225	0.134
5. I felt like a marionette.		0.404	0.405	-0.112	
58. Things around me appeared smaller or larger.	0.224	0.135	0.353		
55. I stayed frozen in a very unnatural position for quite a long time.		0.266	0.318		0.115
**f4**					
65. I experienced an all-embracing love.	-0.136	-0.141		0.862	
61. Everything around me seemed animated.				0.685	
7. I enjoyed boundless pleasure.		-0.156		0.678	0.209
66. My experience had religious aspects.				0.671	-0.348
39. Many things appeared to be breathtakingly beautiful.	0.238			0.666	
60. I experienced a profound peace in myself.		-0.268	0.117	0.659	
27. I experienced a touch of eternity.			0.260	0.614	-0.282
26. I felt unusual powers in myself.				0.595	-0.177
21. It seemed to me that my environment and I were one.		-0.125	0.345	0.589	
34. I felt very profound.		0.198		0.584	
56. I experienced a kind of awe.		0.133		0.584	-0.147
10. Everything seemed to unify into an oneness.			0.231	0.583	-0.189
46. I gained clarity into connections that puzzled me before.		0.187		0.578	-0.146
52. I had very original thoughts.				0.574	
28. Conflicts and contradictions seemed to dissolve.			0.284	0.553	-0.209
37. Objects around me engaged me emotionally much more than usual.		0.115		0.546	0.328
6. I had the feeling of being connected to a superior power.				0.535	-0.384
50. I felt totally free and released from all responsibilities.		-0.249	0.352	0.521	
9. I felt I was being transformed forever in a marvelous way.		0.185	0.103	0.507	
22. Worries and anxieties of everyday life seemed unimportant to me.		-0.130	0.312	0.497	0.115
2. Bodily sensations were very delightful.				0.487	0.233
40. Things came to mind, which I thought I had forgotten long ago.	0.154	0.133	-0.122	0.465	
1. I felt like I was in a fantastic other world.	0.156		0.271	0.419	
18. Things around me had a new strange meaning for me.		0.214	0.248	0.409	0.228
17. Everyday things gained a special meaning.		0.184	0.169	0.396	0.323
64. I was able to remember certain events unusually clearly.	0.221			0.341	
**f5**					
45. I was not able to complete a thought, my thought repeatedly became disconnected.	0.167	0.218	0.296		0.382
47. Many things seemed unbelievably funny to me.	0.266			0.323	0.372

	f1	f2	f3	f4	f5	f6
**f1**						
66. My experience had religious aspects.	0.815					-0.224
27. I experienced a touch of eternity.	0.754			0.224		-0.103
65. I experienced an all-embracing love.	0.740		-0.130	-0.109		0.215
6. I had the feeling of being connected to a superior power.	0.726					-0.260
10. Everything seemed to unify into an oneness.	0.648			0.186		
28. Conflicts and contradictions seemed to dissolve.	0.644			0.238	0.100	
26. I felt unusual powers in myself.	0.631					
56. I experienced a kind of awe.	0.619		0.113			
21. It seemed to me that my environment and I were one.	0.615		-0.102	0.292		0.108
46. I gained clarity into connections that puzzled me before.	0.596	0.116	0.161			
61. Everything around me seemed animated.	0.565	-0.100				0.260
60. I experienced a profound peace in myself.	0.561		-0.242			0.242
34. I felt very profound.	0.552	0.112	0.185			
9. I felt I was being transformed forever in a marvelous way.	0.504		0.180			
39. Many things appeared to be breathtakingly beautiful.	0.504	0.168			0.130	0.303
35. I experienced past, present and future as an oneness.	0.463			0.411		
7. I enjoyed boundless pleasure.	0.458		-0.118			0.409
31. The world appeared to me beyond good and evil.	0.452		0.113	0.380		
50. I felt totally free and released from all responsibilities.	0.440		-0.205	0.297		0.288
52. I had very original thoughts.	0.436	0.146				0.230
40. Things came to mind, which I thought I had forgotten long ago.	0.397	0.214	0.112	-0.115		
22. Worries and anxieties of everyday life seemed unimportant to me.	0.396			0.256		0.291
1. I felt like I was in a fantastic other world.	0.371	0.174		0.238		0.203
64. I was able to remember certain events unusually clearly.	0.264	0.240				0.147
**f2**						
13. I saw colors before me in total darkness or with closed eyes.		0.772			0.197	
8. I saw regular patterns in complete darkness or with closed eyes.		0.715			0.168	
25. I saw scenes rolling by in total darkness or with my eyes closed.	0.135	0.654		0.102		
20. I saw lights or flashes of light in total darkness or with closed eyes.		0.543			0.116	
4. I saw things that I knew were not real.		0.479		0.118		0.142
49. I could see pictures from my past or fantasy extremely clearly.	0.231	0.471				
57. My imagination was extremely vivid.	0.261	0.446		0.100		0.236
**f3**						
38. I felt threatened.		-0.112	0.852			
29. I was afraid without being able to say exactly why.			0.821			
12. I felt tormented.			0.803		0.104	
63. I had the feeling something horrible would happen.			0.777			
32. I experienced my surroundings as strange and weird.			0.690	0.163		
54. I was afraid to lose my self-control.			0.673	0.187		
19. I was afraid that the state I was in would last forever.			0.672			
30. I experienced everything terrifyingly distorted.			0.668	0.148		
3. I felt surrendered to dark powers.		-0.114	0.564		0.143	
36. I experienced an unbearable emptiness.			0.514			
59. Time passed tormentingly slow			0.487			
5. I felt like a marionette.			0.428	0.363		
62. Everything around me was happening so fast that I no longer could follow what was going on.		0.164	0.362	0.223		0.137
55. I stayed frozen in a very unnatural position for quite a long time.			0.287	0.285		0.125
**f4**						
41. My body seemed to me numb, dead and weird.			0.211	0.674		-0.112
15. It seemed to me as though I did not have a body anymore.	0.356			0.649		-0.221
42. I had the feeling of being outside of my body.	0.327			0.641		-0.186
33. I felt as though I were paralyzed.			0.341	0.530		
43. I felt as though I were floating.	0.345			0.509		
48. The boundaries between myself and my surroundings seemed to blur.	0.364			0.467		
53. I had the feeling that I no longer had a will of my own.			0.363	0.465		
24. I had difficulty in distinguishing important from unimportant things.			0.282	0.454		0.270
44. I felt isolated from everything and everyone.			0.357	0.447		0.130
23. Like in a dream, time and space were changed.	0.203	0.279		0.388		0.195
16. I had difficulty making even the smallest decision.			0.348	0.379		0.209
58. Things around me appeared smaller or larger.			0.176	0.314	0.159	0.160
**f5**						
14. The shapes of things seemed to change by sounds and noises.		0.193			0.802	
51. The colors of things seemed to be changed by sounds and noises.		0.188			0.759	
11. Noises seemed to influence what I saw.	0.111				0.741	
**f6**						
37. Objects around me engaged me emotionally much more than usual.	0.232		0.196			0.544
17. Everyday things gained a special meaning.	0.162		0.237	0.124		0.452
47. Many things seemed unbelievably funny to me.		0.298				0.443
2. Bodily sensations were very delightful.	0.267					0.403
45. I was not able to complete a thought, my thought repeatedly became disconnected.	-0.190	0.184	0.262	0.262		0.386
18. Things around me had a new strange meaning for me.	0.249		0.261	0.196		0.359

	f1	f2	f3	f4	f5	f6	f7
**f1**							
13. I saw colors before me in total darkness or with closed eyes.	0.870						
8. I saw regular patterns in complete darkness or with closed eyes.	0.811		0.107				
25. I saw scenes rolling by in total darkness or with my eyes closed.	0.655	0.109		0.149			
20. I saw lights or flashes of light in total darkness or with closed eyes.	0.600		0.102				
4. I saw things that I knew were not real.	0.501	0.145				0.142	
49. I could see pictures from my past or fantasy extremely clearly.	0.428		-0.163	0.335	-0.132	0.140	
57. My imagination was extremely vivid.	0.399	0.143	-0.100	0.224	0.103	0.255	
**f2**							
41. My body seemed to me numb, dead and weird.		0.737			-0.185		
42. I had the feeling of being outside of my body.		0.682		0.299		-0.133	
15. It seemed to me as though I did not have a body anymore.		0.676		0.270		-0.213	
33. I felt as though I were paralyzed.		0.588	0.237		-0.155		
43. I felt as though I were floating.		0.538		0.200	0.135		
24. I had difficulty in distinguishing important from unimportant things.		0.523	0.189		0.135	0.211	
53. I had the feeling that I no longer had a will of my own.		0.522	0.286				
44. I felt isolated from everything and everyone.		0.516	0.216			0.174	
48. The boundaries between myself and my surroundings seemed to blur.		0.516		0.259			
35. I experienced past, present and future as an oneness.		0.449		0.355			
16. I had difficulty making even the smallest decision.		0.439	0.266			0.167	
23. Like in a dream, time and space were changed.	0.276	0.431			0.202	0.116	
31. The world appeared to me beyond good and evil.		0.417		0.325	0.130		
5. I felt like a marionette.		0.411	0.354				
58. Things around me appeared smaller or larger.	0.119	0.364				0.178	0.140
55. I stayed frozen in a very unnatural position for quite a long time.		0.340	0.176			0.175	
62. Everything around me was happening so fast that I no longer could follow what was going on.	0.124	0.278	0.254			0.185	
1. I felt like I was in a fantastic other world.	0.179	0.270		0.191	0.266	0.118	
**f3**							
38. I felt threatened.			0.850				
29. I was afraid without being able to say exactly why.			0.841				
63. I had the feeling something horrible would happen.			0.796				
12. I felt tormented.			0.761		-0.122		
19. I was afraid that the state I was in would last forever.			0.679				-0.101
32. I experienced my surroundings as strange and weird.		0.204	0.651				
30. I experienced everything terrifyingly distorted.		0.189	0.623				
54. I was afraid to lose my self-control.		0.237	0.608				
3. I felt surrendered to dark powers.			0.548				0.134
36. I experienced an unbearable emptiness.		0.115	0.424	0.130	-0.118	0.104	
59. Time passed tormentingly slow			0.405		-0.111	0.126	
**f4**							
66. My experience had religious aspects.				0.797		-0.104	
46. I gained clarity into connections that puzzled me before.				0.715	-0.134	0.180	
6. I had the feeling of being connected to a superior power.				0.678		-0.181	
27. I experienced a touch of eternity.		0.251		0.626	0.138		
56. I experienced a kind of awe.				0.562			
40. Things came to mind, which I thought I had forgotten long ago.	0.113			0.545	-0.151	0.301	
34. I felt very profound.			0.133	0.539		0.138	
26. I felt unusual powers in myself.				0.534	0.137		
28. Conflicts and contradictions seemed to dissolve.		0.269		0.514	0.151		
10. Everything seemed to unify into an oneness.		0.213		0.499	0.189		
52. I had very original thoughts.				0.492		0.365	
9. I felt I was being transformed forever in a marvelous way.		0.121	0.120	0.461		0.115	
61. Everything around me seemed animated.	-0.103			0.376	0.315	0.196	
64. I was able to remember certain events unusually clearly.	0.179		-0.117	0.325		0.254	
**f5**							
60. I experienced a profound peace in myself.					0.732		
7. I enjoyed boundless pleasure.	0.103				0.669	0.130	
22. Worries and anxieties of everyday life seemed unimportant to me.		0.267			0.602		
50. I felt totally free and released from all responsibilities.		0.308			0.600		
65. I experienced an all-embracing love.		-0.119		0.411	0.540		
39. Many things appeared to be breathtakingly beautiful.	0.229			0.237	0.449	0.154	
21. It seemed to me that my environment and I were one.		0.307		0.307	0.423		
2. Bodily sensations were very delightful.					0.372	0.266	
**f6**							
37. Objects around me engaged me emotionally much more than usual.	-0.107			0.166	0.190	0.568	0.115
17. Everyday things gained a special meaning.		0.207		0.158		0.527	
18. Things around me had a new strange meaning for me.		0.286		0.264		0.467	
45. I was not able to complete a thought, my thought repeatedly became disconnected.	0.157	0.332	0.118	-0.185		0.383	
47. Many things seemed unbelievably funny to me.	0.287				0.245	0.371	
**f7**							
14. The shapes of things seemed to change by sounds and noises.	0.458						0.684
11. Noises seemed to influence what I saw.	0.322						0.641
51. The colors of things seemed to be changed by sounds and noises.	0.453						0.640

	f1	f2	f3	f4	f5	f6	f7	f8
**f1**								
13. I saw colors before me in total darkness or with closed eyes.	0.902							
8. I saw regular patterns in complete darkness or with closed eyes.	0.839							
20. I saw lights or flashes of light in total darkness or with closed eyes.	0.536							0.105
25. I saw scenes rolling by in total darkness or with my eyes closed.	0.484					-0.195		0.436
4. I saw things that I knew were not real.	0.442	0.149				0.128		0.115
**f2**								
41. My body seemed to me numb, dead and weird.		0.732	0.105		-0.186			
15. It seemed to me as though I did not have a body anymore.		0.686		0.244		-0.186		
42. I had the feeling of being outside of my body.		0.677		0.225		-0.158		
33. I felt as though I were paralyzed.		0.573	0.268		-0.134			
43. I felt as though I were floating.		0.529		0.134	0.169	-0.104		
48. The boundaries between myself and my surroundings seemed to blur.		0.527		0.276				
24. I had difficulty in distinguishing important from unimportant things.		0.521	0.196		0.121	0.215		
53. I had the feeling that I no longer had a will of my own.		0.515	0.302					
44. I felt isolated from everything and everyone.		0.503	0.242			0.159		
35. I experienced past, present and future as an oneness.		0.452		0.323				
16. I had difficulty making even the smallest decision.		0.439	0.260			0.184		
23. Like in a dream, time and space were changed.	0.219	0.425			0.217			0.123
31. The world appeared to me beyond good and evil.		0.421		0.311	0.129			
5. I felt like a marionette.		0.415	0.336					-0.113
58. Things around me appeared smaller or larger.		0.355				0.159	0.163	
55. I stayed frozen in a very unnatural position for quite a long time.		0.325	0.209			0.130		0.118
1. I felt like I was in a fantastic other world.	0.181	0.282		0.227	0.236	0.119		
**f3**								
29. I was afraid without being able to say exactly why.			0.865					
38. I felt threatened.			0.848					
63. I had the feeling something horrible would happen.			0.835			-0.118		
12. I felt tormented.			0.753		-0.132			
19. I was afraid that the state I was in would last forever.			0.680				-0.103	
30. I experienced everything terrifyingly distorted.		0.169	0.650					
32. I experienced my surroundings as strange and weird.		0.196	0.650					
54. I was afraid to lose my self-control.		0.219	0.631					
3. I felt surrendered to dark powers.			0.544				0.136	
36. I experienced an unbearable emptiness.			0.448	0.109				
59. Time passed tormentingly slow			0.415			0.104		
62. Everything around me was happening so fast that I no longer could follow what was going on.		0.259	0.294			0.122		0.114
**f4**								
66. My experience had religious aspects.				0.783		-0.130		
46. I gained clarity into connections that puzzled me before.				0.668	-0.110			0.152
6. I had the feeling of being connected to a superior power.				0.666		-0.187		
27. I experienced a touch of eternity.		0.276		0.646				
56. I experienced a kind of awe.				0.570				
34. I felt very profound.			0.119	0.544				
26. I felt unusual powers in myself.				0.536	0.124			
28. Conflicts and contradictions seemed to dissolve.		0.287		0.524	0.122			
10. Everything seemed to unify into an oneness.		0.228		0.505	0.166			
9. I felt I was being transformed forever in a marvelous way.		0.132	0.106	0.472				
52. I had very original thoughts.				0.469		0.273		0.179
40. Things came to mind, which I thought I had forgotten long ago.				0.423		0.144		0.384
61. Everything around me seemed animated.				0.421	0.295	0.179		
**f5**								
60. I experienced a profound peace in myself.					0.761	-0.121		
7. I enjoyed boundless pleasure.					0.705			0.103
50. I felt totally free and released from all responsibilities.		0.300			0.631			
22. Worries and anxieties of everyday life seemed unimportant to me.		0.282			0.565			-0.143
65. I experienced an all-embracing love.		-0.116		0.397	0.556			
39. Many things appeared to be breathtakingly beautiful.	0.218			0.288	0.423	0.146	0.102	
2. Bodily sensations were very delightful.					0.419	0.192		0.126
21. It seemed to me that my environment and I were one.		0.320		0.326	0.396			
**f6**								
37. Objects around me engaged me emotionally much more than usual.				0.289	0.147	0.574		
17. Everyday things gained a special meaning.		0.217		0.255		0.523		
18. Things around me had a new strange meaning for me.		0.312		0.406		0.515		
45. I was not able to complete a thought, my thought repeatedly became disconnected.		0.309	0.163	-0.200		0.318		0.178
47. Many things seemed unbelievably funny to me.	0.244				0.264	0.313		0.136
**f7**								
14. The shapes of things seemed to change by sounds and noises.	0.304						0.745	
51. The colors of things seemed to be changed by sounds and noises.	0.276						0.710	
11. Noises seemed to influence what I saw.	0.174						0.694	
**f8**								
49. I could see pictures from my past or fantasy extremely clearly.	0.155							0.704
64. I was able to remember certain events unusually clearly.				0.139				0.525
57. My imagination was extremely vivid.	0.263	0.123		0.114	0.167	0.122		0.369

	f1	f2	f3	f4	f5	f6	f7	f8	f9
**f1**									
13. I saw colors before me in total darkness or with closed eyes.	0.911								
8. I saw regular patterns in complete darkness or with closed eyes.	0.834								
20. I saw lights or flashes of light in total darkness or with closed eyes.	0.538							0.103	
4. I saw things that I knew were not real.	0.425		0.115			0.167		0.142	
**f2**									
29. I was afraid without being able to say exactly why.		0.858							
63. I had the feeling something horrible would happen.		0.855				-0.194			0.171
38. I felt threatened.		0.853							
12. I felt tormented.		0.757			-0.138				
19. I was afraid that the state I was in would last forever.		0.682					-0.103		
32. I experienced my surroundings as strange and weird.		0.656	0.188						
30. I experienced everything terrifyingly distorted.		0.655	0.179						
54. I was afraid to lose my self-control.		0.630	0.213						
3. I felt surrendered to dark powers.		0.551		0.134			0.141		-0.276
36. I experienced an unbearable emptiness.		0.451	0.145						0.258
59. Time passed tormentingly slow		0.420							0.171
62. Everything around me was happening so fast that I no longer could follow what was going on.	0.104	0.291	0.268			0.108			0.112
**f3**									
41. My body seemed to me numb, dead and weird.		0.103	0.745		-0.179				
42. I had the feeling of being outside of my body.			0.735	0.190		-0.204			0.131
15. It seemed to me as though I did not have a body anymore.			0.700	0.231		-0.183			
43. I felt as though I were floating.			0.568		0.190	-0.140			
33. I felt as though I were paralyzed.		0.268	0.548		-0.141				
48. The boundaries between myself and my surroundings seemed to blur.			0.516	0.259					
44. I felt isolated from everything and everyone.		0.241	0.496			0.158			
53. I had the feeling that I no longer had a will of my own.		0.300	0.486						
24. I had difficulty in distinguishing important from unimportant things.		0.196	0.480		0.113	0.255			
35. I experienced past, present and future as an oneness.			0.456	0.300	0.108				
31. The world appeared to me beyond good and evil.			0.430	0.298	0.138				
23. Like in a dream, time and space were changed.	0.213		0.418		0.226			0.123	
16. I had difficulty making even the smallest decision.		0.259	0.405			0.217			
58. Things around me appeared smaller or larger.		0.104	0.372			0.116	0.155		0.150
5. I felt like a marionette.		0.335	0.367		-0.103	0.145			-0.179
55. I stayed frozen in a very unnatural position for quite a long time.		0.207	0.292			0.171		0.136	
1. I felt like I was in a fantastic other world.	0.172		0.259	0.222	0.241	0.140			
**f4**									
66. My experience had religious aspects.				0.808					
6. I had the feeling of being connected to a superior power.				0.704		-0.137			-0.140
27. I experienced a touch of eternity.			0.268	0.639	0.109				
46. I gained clarity into connections that puzzled me before.				0.637					0.289
56. I experienced a kind of awe.				0.555					
26. I felt unusual powers in myself.				0.550	0.122				
28. Conflicts and contradictions seemed to dissolve.			0.267	0.532	0.119				
34. I felt very profound.		0.118		0.518					0.199
10. Everything seemed to unify into an oneness.			0.208	0.511	0.167				
9. I felt I was being transformed forever in a marvelous way.		0.102	0.108	0.479		0.109			
52. I had very original thoughts.				0.447		0.244		0.118	0.188
61. Everything around me seemed animated.				0.389	0.314	0.133			0.142
40. Things came to mind, which I thought I had forgotten long ago.				0.372				0.301	0.362
**f5**									
60. I experienced a profound peace in myself.					0.769	-0.139			
7. I enjoyed boundless pleasure.					0.700			0.127	
50. I felt totally free and released from all responsibilities.			0.309		0.628				
65. I experienced an all-embracing love.				0.382	0.569				
22. Worries and anxieties of everyday life seemed unimportant to me.			0.267		0.558			-0.118	
2. Bodily sensations were very delightful.					0.428	0.162			0.121
39. Many things appeared to be breathtakingly beautiful.	0.226			0.282	0.427	0.140			
21. It seemed to me that my environment and I were one.			0.310	0.321	0.401				
**f6**									
18. Things around me had a new strange meaning for me.			0.250	0.401		0.541			
17. Everyday things gained a special meaning.			0.171	0.245		0.532			
37. Objects around me engaged me emotionally much more than usual.				0.251	0.166	0.520			0.204
45. I was not able to complete a thought, my thought repeatedly became disconnected.		0.162	0.265	-0.193		0.359		0.195	
47. Many things seemed unbelievably funny to me.	0.236				0.257	0.333		0.131	
**f7**									
14. The shapes of things seemed to change by sounds and noises.	0.387						0.711		
51. The colors of things seemed to be changed by sounds and noises.	0.353						0.680		
11. Noises seemed to influence what I saw.	0.237						0.670		
**f8**									
49. I could see pictures from my past or fantasy extremely clearly.								0.795	
64. I was able to remember certain events unusually clearly.				0.103				0.490	0.203
25. I saw scenes rolling by in total darkness or with my eyes closed.	0.435					-0.126		0.486	
57. My imagination was extremely vivid.	0.241		0.109	0.102	0.170	0.142		0.361	

	f1	f2	f3	f4	f5	f6	f7	f8	f9	f10
**f1**										
13. I saw colors before me in total darkness or with closed eyes.	0.821				0.169					
8. I saw regular patterns in complete darkness or with closed eyes.	0.753				0.147					
20. I saw lights or flashes of light in total darkness or with closed eyes.	0.482				0.125					
4. I saw things that I knew were not real.	0.388						0.234		0.154	
**f2**										
66. My experience had religious aspects.		0.817					-0.132			
6. I had the feeling of being connected to a superior power.		0.703					-0.165			
46. I gained clarity into connections that puzzled me before.		0.613								0.308
27. I experienced a touch of eternity.		0.594		0.311		0.104				
56. I experienced a kind of awe.		0.588						0.151		0.138
26. I felt unusual powers in myself.		0.553				0.128				
28. Conflicts and contradictions seemed to dissolve.		0.527		0.198	0.105	0.125		0.103		
34. I felt very profound.		0.506	0.115							0.222
9. I felt I was being transformed forever in a marvelous way.		0.499						0.180		
10. Everything seemed to unify into an oneness.		0.496		0.185		0.167				
52. I had very original thoughts.		0.452					0.197		0.102	0.220
61. Everything around me seemed animated.		0.402				0.315				0.179
**f3**										
63. I had the feeling something horrible would happen.		-0.118	0.865				-0.103			0.139
38. I felt threatened.			0.845							
29. I was afraid without being able to say exactly why.			0.776					0.146		
12. I felt tormented.			0.695	-0.152		-0.133		0.106		
19. I was afraid that the state I was in would last forever.	0.105		0.688		-0.135					
32. I experienced my surroundings as strange and weird.			0.572					0.219		
54. I was afraid to lose my self-control.			0.526					0.280		
30. I experienced everything terrifyingly distorted.			0.521					0.335		0.104
3. I felt surrendered to dark powers.	-0.116	0.134	0.486		0.125			0.140		-0.249
59. Time passed tormentingly slow			0.421				0.122			0.141
36. I experienced an unbearable emptiness.			0.415					0.101		0.234
**f4**										
15. It seemed to me as though I did not have a body anymore.		0.138		0.736						
42. I had the feeling of being outside of my body.		0.133		0.665				0.181		0.125
43. I felt as though I were floating.				0.569		0.177				
41. My body seemed to me numb, dead and weird.				0.486		-0.176		0.434		
35. I experienced past, present and future as an oneness.		0.252		0.428		0.104				
48. The boundaries between myself and my surroundings seemed to blur.		0.216		0.416			0.104	0.152		
23. Like in a dream, time and space were changed.	0.221			0.410		0.225	0.225		0.145	
31. The world appeared to me beyond good and evil.		0.284		0.326		0.138		0.184		
58. Things around me appeared smaller or larger.		-0.128	0.123	0.325	0.160		0.226			0.121
**f5**										
14. The shapes of things seemed to change by sounds and noises.	0.139				0.852					
51. The colors of things seemed to be changed by sounds and noises.	0.119				0.801					
11. Noises seemed to influence what I saw.					0.759					
**f6**										
60. I experienced a profound peace in myself.				0.121		0.759	-0.154			
7. I enjoyed boundless pleasure.						0.697			0.127	
50. I felt totally free and released from all responsibilities.				0.255		0.623				
65. I experienced an all-embracing love.		0.401				0.563				0.113
22. Worries and anxieties of everyday life seemed unimportant to me.				0.189		0.557			-0.110	-0.100
2. Bodily sensations were very delightful.						0.432	0.138			0.140
39. Many things appeared to be breathtakingly beautiful.	0.183	0.263			0.120	0.422	0.163	-0.151		
21. It seemed to me that my environment and I were one.		0.297		0.302		0.396				
1. I felt like I was in a fantastic other world.	0.170	0.179		0.240		0.242	0.208			
**f7**										
18. Things around me had a new strange meaning for me.		0.366		0.113			0.626			
17. Everyday things gained a special meaning.		0.203					0.583			
37. Objects around me engaged me emotionally much more than usual.		0.220				0.165	0.551			0.181
47. Many things seemed unbelievably funny to me.	0.213			-0.155		0.269	0.294		0.125	
**f8**										
33. I felt as though I were paralyzed.				0.200		-0.120		0.596		
62. Everything around me was happening so fast that I no longer could follow what was going on.	0.102							0.592		0.211
16. I had difficulty making even the smallest decision.							0.104	0.587		
53. I had the feeling that I no longer had a will of my own.			0.114	0.162				0.533		
44. I felt isolated from everything and everyone.				0.180			0.102	0.513		
45. I was not able to complete a thought, my thought repeatedly became disconnected.		-0.150					0.259	0.486	0.176	
5. I felt like a marionette.			0.176					0.453		-0.156
24. I had difficulty in distinguishing important from unimportant things.				0.169		0.136	0.214	0.453		
55. I stayed frozen in a very unnatural position for quite a long time.							0.116	0.376	0.121	
**f9**										
49. I could see pictures from my past or fantasy extremely clearly.									0.806	
25. I saw scenes rolling by in total darkness or with my eyes closed.	0.417								0.491	
64. I was able to remember certain events unusually clearly.		0.126							0.476	0.219
57. My imagination was extremely vivid.	0.232					0.171	0.166		0.358	
**f10**										
40. Things came to mind, which I thought I had forgotten long ago.		0.365							0.286	0.369

	f1	f2	f3	f4	f5	f6	f7	f8	f9	f10	f11
**f2**											
13. I saw colors before me in total darkness or with closed eyes.		0.811				0.184					
8. I saw regular patterns in complete darkness or with closed eyes.		0.751				0.156					
20. I saw lights or flashes of light in total darkness or with closed eyes.		0.485				0.127					
4. I saw things that I knew were not real.		0.395					0.237				0.145
**f3**											
66. My experience had religious aspects.	-0.169		0.774				-0.125				
46. I gained clarity into connections that puzzled me before.	0.206		0.695								
6. I had the feeling of being connected to a superior power.	-0.239		0.647				-0.157				
56. I experienced a kind of awe.			0.605						0.162		
27. I experienced a touch of eternity.			0.573					0.222		0.194	
34. I felt very profound.			0.552	0.113							
26. I felt unusual powers in myself.	-0.155		0.533		0.147						
28. Conflicts and contradictions seemed to dissolve.			0.532			0.118				0.463	
52. I had very original thoughts.	0.127		0.510				0.202				0.106
9. I felt I was being transformed forever in a marvelous way.			0.491						0.178		
40. Things came to mind, which I thought I had forgotten long ago.	0.293		0.479								0.293
10. Everything seemed to unify into an oneness.			0.474		0.103					0.267	
61. Everything around me seemed animated.			0.440		0.337						
31. The world appeared to me beyond good and evil.			0.300					0.177	0.173	0.293	
**f4**											
63. I had the feeling something horrible would happen.	0.171			0.880			-0.116				
38. I felt threatened.				0.842							
29. I was afraid without being able to say exactly why.				0.779				-0.130	0.142		
19. I was afraid that the state I was in would last forever.		0.108		0.700		-0.131					
12. I felt tormented.				0.694	-0.136			-0.140	0.102		
32. I experienced my surroundings as strange and weird.				0.568					0.226		
54. I was afraid to lose my self-control.				0.526					0.280		
30. I experienced everything terrifyingly distorted.				0.521					0.345		
3. I felt surrendered to dark powers.	-0.405	-0.106		0.467		0.103			0.142		
36. I experienced an unbearable emptiness.	0.259		0.122	0.434							
59. Time passed tormentingly slow	0.169			0.427			0.114				
**f5**											
60. I experienced a profound peace in myself.					0.754		-0.144				
7. I enjoyed boundless pleasure.					0.669					0.110	0.115
65. I experienced an all-embracing love.			0.419		0.567						
50. I felt totally free and released from all responsibilities.					0.555					0.323	
2. Bodily sensations were very delightful.					0.467		0.152				
22. Worries and anxieties of everyday life seemed unimportant to me.					0.462					0.410	-0.100
39. Many things appeared to be breathtakingly beautiful.		0.190	0.258		0.442	0.109	0.180		-0.142		
1. I felt like I was in a fantastic other world.		0.177	0.165		0.235		0.216	0.222			
**f6**											
14. The shapes of things seemed to change by sounds and noises.		0.133				0.844					
51. The colors of things seemed to be changed by sounds and noises.		0.113				0.794					
11. Noises seemed to influence what I saw.						0.751			-0.114	0.152	
**f7**											
18. Things around me had a new strange meaning for me.			0.354				0.623	0.112			
17. Everyday things gained a special meaning.			0.227				0.586				
37. Objects around me engaged me emotionally much more than usual.	0.123		0.280		0.169		0.553				
47. Many things seemed unbelievably funny to me.		0.212			0.249		0.304	-0.163			0.121
**f8**											
15. It seemed to me as though I did not have a body anymore.	-0.112		0.104					0.727			
43. I felt as though I were floating.					0.266			0.664		-0.169	
42. I had the feeling of being outside of my body.			0.164					0.636	0.199		
41. My body seemed to me numb, dead and weird.					-0.182			0.445	0.426		
48. The boundaries between myself and my surroundings seemed to blur.			0.207				0.112	0.385	0.155		
23. Like in a dream, time and space were changed.		0.228			0.211		0.227	0.368			0.136
58. Things around me appeared smaller or larger.				0.111		0.156	0.226	0.360			
35. I experienced past, present and future as an oneness.			0.263					0.303		0.259	
**f9**											
62. Everything around me was happening so fast that I no longer could follow what was going on.									0.619	-0.139	
33. I felt as though I were paralyzed.					-0.123			0.198	0.585		
16. I had difficulty making even the smallest decision.							0.111		0.564	0.141	
44. I felt isolated from everything and everyone.							0.111	0.204	0.515		
53. I had the feeling that I no longer had a will of my own.				0.123					0.505	0.217	
45. I was not able to complete a thought, my thought repeatedly became disconnected.			-0.138				0.274		0.475		0.167
5. I felt like a marionette.	-0.167			0.174	-0.107				0.430		
24. I had difficulty in distinguishing important from unimportant things.							0.214		0.429	0.253	
55. I stayed frozen in a very unnatural position for quite a long time.	-0.105						0.127		0.376		0.111
**f10**											
21. It seemed to me that my environment and I were one.			0.272		0.318			0.158		0.328	
**f11**											
49. I could see pictures from my past or fantasy extremely clearly.			0.104								0.792
25. I saw scenes rolling by in total darkness or with my eyes closed.		0.430									0.481
64. I was able to remember certain events unusually clearly.	0.178		0.200								0.472
57. My imagination was extremely vivid.		0.239	0.124		0.172		0.172				0.348
