# Supplementary material for: Psychometric Evaluation of the Altered States of Consciousness Rating Scale (OAV)
Source: PLoS One. 2010 Aug 31;5(8):e12412. doi: 10.1371/journal.pone.0012412 (PMC2930851; doi:10.1371/journal.pone.0012412)
Supplement: Table S7 — Y-standardized regression coeffcients [and 95% confidence intervals] of MIMIC model with dose predictors and DIF. (0.05 MB PDF) [file pone.0012412.s009.pdf]

Supplementary Table S7. Y-standardized regression coeffcients [and 95% confidence intervals] of MIMIC model with dose predictors and DIF.

Factor	PET	Female	OAV	Psilo LD	Psilo MD 1	Psilo MD 2	Psilo HD	Ket MD	Ket HD
Experience of unity	**0.25**	**0.25**	0.01	**-0.37**	0.31	0.19	**0.55**	0.19	**0.54**
	[0.03, 0.47]	[0.03, 0.47]	[-0.25, 0.27]	[-0.69, -0.05]	[-0.08, 0.69]	[-0.06, 0.45]	[0.11, 0.99]	[-0.28, 0.66]	[0.24, 0.83]
Spiritual experience	**0.26**	0.09	0.14	0.06	0.41	**0.46**	**0.62**	0.10	**0.51**
	[0.01, 0.51]	[-0.18, 0.36]	[-0.17, 0.45]	[-0.30, 0.41]	[-0.03, 0.86]	[0.20, 0.71]	[0.20, 1.05]	[-0.37, 0.57]	[0.21, 0.81]
Blissful state	0.18	0.05	0.13	**-0.57**	0.04	-0.06	0.16	**-0.66**	**-0.50**
	[-0.06, 0.43]	[-0.16, 0.27]	[-0.15, 0.40]	[-0.94, -0.20]	[-0.39, 0.47]	[-0.34, 0.23]	[-0.28, 0.59]	[-1.11, -0.21]	[-0.80, -0.19]
Insightfulness	**0.26**	-0.16	**0.35**	0.21	**0.78**	**0.61**	**1.12**	0.38	0.29
	[0.01, 0.51]	[-0.39, 0.07]	[0.05, 0.64]	[-0.11, 0.54]	[0.37, 1.19]	[0.34, 0.88]	[0.62, 1.61]	[-0.17, 0.93]	[-0.01, 0.59]
Disembodiment	**0.55**	**0.24**	-0.04	-0.21	0.33	0.06	**0.57**	**0.62**	**0.93**
	[0.33, 0.77]	[0.02, 0.45]	[-0.27, 0.18]	[-0.50, 0.08]	[-0.03, 0.69]	[-0.16, 0.28]	[0.14, 0.99]	[0.13, 1.11]	[0.67, 1.19]
Impaired control and cognition	0.15	**0.38**	-0.16	**-0.28**	**0.38**	**0.37**	**0.74**	**0.95**	**0.93**
	[-0.07, 0.38]	[0.14, 0.62]	[-0.39, 0.07]	[-0.54, -0.02]	[0.06, 0.70]	[0.14, 0.60]	[0.31, 1.18]	[0.44, 1.46]	[0.65, 1.20]
Anxiety	**0.32**	0.05	-0.16	-0.07	0.11	**0.24**	**0.55**	-0.10	**0.56**
	[0.08, 0.56]	[-0.15, 0.25]	[-0.39, 0.06]	[-0.33, 0.18]	[-0.15, 0.37]	[0.05, 0.43]	[0.03, 1.07]	[-0.56, 0.37]	[0.29, 0.84]
Complex imagery	**0.52**	0.12	-0.04	**0.44**	**1.16**	**0.83**	**1.39**	0.20	**0.69**
	[0.30, 0.75]	[-0.11, 0.35]	[-0.28, 0.20]	[0.11, 0.77]	[0.81, 1.51]	[0.61, 1.04]	[0.98, 1.79]	[-0.27, 0.67]	[0.42, 0.96]
Elementary imagery	**0.28**	0.05	-0.01	**0.75**	**1.46**	**1.45**	**1.71**	**0.65**	**0.82**
	[0.10, 0.47]	[-0.13, 0.23]	[-0.20, 0.19]	[0.49, 1.02]	[1.18, 1.74]	[1.25, 1.64]	[1.37, 2.05]	[0.27, 1.03]	[0.58, 1.07]
Audio-visual synesthesiae	-0.10	0.06	0.16	**0.35**	**0.86**	**0.90**	**1.31**	**0.60**	**0.62**
	[-0.31, 0.11]	[-0.16, 0.28]	[-0.08, 0.39]	[0.09, 0.61]	[0.52, 1.20]	[0.69, 1.10]	[0.92, 1.71]	[0.20, 1.00]	[0.35, 0.89]
Changed meaning of percepts	**0.37**	-0.04	0.20	-0.04	**0.85**	**0.53**	**0.95**	0.20	0.17
	[0.14, 0.59]	[-0.30, 0.22]	[-0.06, 0.47]	[-0.36, 0.29]	[0.48, 1.21]	[0.27, 0.79]	[0.50, 1.39]	[-0.26, 0.66]	[-0.16, 0.49]

Note. Significant regression coefficients ($p < .05$) are in boldface. By convention, y-standardized regression coefficients of dummy coded variables of the sizes 0.2, 0.5, and 0.8 indicate small, medium, and large effect sizes, respectively. MIMIC = multiple indicators multiple causes; DIF = differential item functioning; PET = positron emission tomography (0 = no PET, 1 = PET); Female (0 = male, 1 = female); OAV = Altered state of consciousness rating scale version (0 = OAV, 1 = 5D-ASC); Psilo LD (0 = all drug conditions except 115 µg/kg psilocybin, 1 = 115 µg/kg psilocybin); Psilo MD 1 (0 = all drug conditions except 215-225 µg/kg psilocybin, 1 = 215-225 µg/kg psilocybin); Psilo MD 2 (0 = all drug conditions except 250-270 µg/kg psilocybin, 1 = 250-270 µg/kg psilocybin); Psilo HD (0 = all drug conditions except 315 µg/kg psilocybin, 1 = 315 µg/kg psilocybin); Ket MD (0 = all drug conditions except 6 µg/kg/min ketamine, 1 = 6 µg/kg/min; Ket HD (0 = all drug conditions except 12 µg/kg/min ketamine, 1 = 12 µg/kg/min ketamine).
