# Supplementary material for: Psychometric Evaluation of the Altered States of Consciousness Rating Scale (OAV)
Source: PLoS One. 2010 Aug 31;5(8):e12412. doi: 10.1371/journal.pone.0012412 (PMC2930851; doi:10.1371/journal.pone.0012412)
Supplement: Methods S1 — (0.05 MB DOC) [file pone.0012412.s010.doc]

**Supplementary Methods S1**

Polychoric correlations estimate the linear associations of latent continuous variables underlying the manifest categorical variables. Because polychoric correlations rest on the assumption that the underlying continuous variables are bivariate normal and that the observed responses are determined by the respondents’ thresholds, which are usually converted to the underlying continuous variable by a non-linear transformation, items were categorized such that the degree of normality of the categorized variables was maximized.

Although polychoric correlations seem to be robust to modest violations of underlying bivariate normality [1], it is generally recommended to test this assumption by the LR-χ^2^ statistic and to evaluate model fit by the root mean square error of approximation [RMSEA; 2]. According to simulation studies, no serious effects of non-normality on the polychoric correlation estimate are expected to occur as long as RMSEA values are below 0.1 [3]. The cut-points that we used in the present study (1, 30, 70, and 90 across all items) produced RMSEA values that slightly exceeded 0.10 in only 2 of 2211 estimated polychoric correlations and therefore should not have resulted in biased parameter estimates. However, it should be noted that polychoric correlations can still be unreliable when estimated on the basis of frequency tables with low expected cell frequencies [3]. Because we used 5 categories and equal cut-points across all items, it was unavoidable that some cells of the frequency tables of item pairs became empty. Although a lower number of categories would have reduced the number of empty cells, it would have also increased the information loss resulting from the item categorization. Hence, for the lack of better alternatives, we had to accept this small degree of potential bias.

Another reason why we decided to cross-check our results by using categorical variable methodology was the occurrence of L-shape patterns in bivariate scatter plots of several OAV item pairs describing experience of opposite affective valence. L-shaped bivariate distributions are well known to occur between strictly unipolar items measuring opposite halves of one underlying bipolar continuum, because giving a positive response in one item implies giving a neutral response in the other item and vice versa [2]. Due to the opposite skew of such items, the size of their correlation is markedly attenuated when estimated by the product-moment correlation. In fact, it can be shown that the negative correlation between two items measuring mutually exclusive parts of one bipolar continuum theoretically cannot exceed -.47 [4]. The only exception to this rule is when the sample is highly polarized [for more details, see 5]. In contrast to the product-moment correlation, the polychoric correlation can correctly estimate a perfect negative correlation when an L-shape pattern is present. Since most factor analyses in the past have relied on product-moment correlations, the above issue has often led to biased estimates of correlations between latent variables or to the unexpected finding of two separate unipolar dimensions when only one bipolar dimension was hypothesized [see 2]. Because the OAV contains strictly unipolar items and because some OAV item pairs of opposite affective valence tended to form L-shape patterns, we hypothesized that the association between these items would become more negative when estimated by polychoric instead of product-moment-correlations and that this would eventually lead to more negative correlations between latent factors for which these items were indicators. Furthermore, we hypothesized that the attenuation of product-moment correlations induced by the L-shape patterns could explain at least in part the unexpected high positive correlation between OAV factors covering experiences of opposite affective valence (eg, OBN with DED) reported in previous factor analyses [6,7].

In the latent factor models, the polychoric correlation matrix of the categorized variables was used in combination with the robust weighted least squares estimator (WLSMV in M*plus*). The WLSMV provides weighted least square parameter estimates using a diagonal weight matrix and robust standard errors and a mean- and variance adjusted χ^2^ [8]. Due to good performance in Monte Carlo studies, the WLSMV is currently the method of choice for the analysis of skewed categorical data in small to moderate samples [9,10].
